# AI enhanced model predictive control for optimizing LPG recovery through integrated computational modeling design of experiments and multivariate regression

**DOI:** 10.1038/s41598-025-13899-z

**Published:** 2025-08-10

**Authors:** Basma Abd El Hakim, Mahmoud Abdel-Halim Abdel-Goad, M. E. Awad, Abeer M. Shoaib

**Affiliations:** 1 Chemical Engineering Department, Faculty of Engineering, Minia University, Minia, Egypt; 2 Petroleum Refining and Petrochemical Engineering Department, Faculty of Petroleum and Mining Engineering, Suez University, Suez, 43512 Egypt

**Keywords:** LPG recovery, Response surface methodology, MPC controller, AI-enhanced MPC, Machine learning, Dynamic control, Chemical engineering, Natural gas

## Abstract

Liquefied Petroleum Gas (LPG) recovery in debutanizer columns presents challenges in balancing operational efficiency and process stability under varying conditions. Conventional control strategies often fail to sustain optimal recovery. This study integrates process modeling and control, using Aspen HYSYS for steady-state simulation and dynamic implementation of model predictive control (MPC). Response surface methodology (RSM) was applied to steady-state simulation results to analyze key process variables. Feed molar flow rate was the most influential factor, while pressure-related variables showed minor but statistically significant effects. The quadratic model and 3D response surfaces confirmed key interactions. A regression decision tree model was developed in MATLAB to support deployment of artificial intelligence-enhanced MPC (AI-enhanced MPC). MPC improved LPG recovery from 99.73 to 99.85%, reduced reboiler duty from 1,557,000 to 1,550,000 kcal/h, and reflux flow from 281.2 to 271 kgmole/h. AI-enhanced MPC further increased recovery to 99.9%, reduced reboiler duty to 1,501,956 kcal/h, condenser duty to 2,415,726 kcal/h, and reflux flow to 262.6 kgmole/h, indicating superior energy efficiency and control precision. Although feed molar flow remained dominant, both control systems regulated its impact via pressure, temperature, and reflux. Product temperature dropped from 49.88 °C to 49.24 °C, and pressure from 12.39 to 11.95 bar, indicating enhanced thermal stability. The novelty of this study lies in integrating RSM with both conventional and AI-enhanced model predictive control, forming a hybrid framework enabling steady-state optimization and dynamic control for improved LPG recovery. The proposed framework supports industrial LPG recovery by improving energy efficiency, product quality, and dynamic stability.

## Introduction

Natural gas (NG) serves as a crucial energy carrier in the 21 st century due to its clean combustion properties and widespread availability^1^. In recent years, the processing of NG for the recovery of natural gas liquids (NGLs) has gained significant attention. The economic advantages of NGL recovery are maximized when the extracted components are marketed separately^2^; among these products, liquefied petroleum gas (LPG), primarily composed of propane and butane. It is a non-toxic, highly flammable fuel with high octane ratings and environmentally friendly characteristics^3^. LPG is widely utilized for residential heating, motor fuel applications, and cooking purposes^4^. Additionally, it is recognized as a crucial transitional fuel in the shift toward sustainable energy and transportation solutions^5^. Given its high market value, selling LPG alongside NG condensates or lower-cost dry gas could significantly diminish its economic benefits^6^. The profitability of LPG recovery is further enhanced by its potential use in internal combustion engines as an alternative to conventional fuels. Studies indicate that gasoline multipoint injection engine systems can be readily adapted to operate with LPG^7,8^. Moreover, LPG remains a preferred choice for residential heating and cooking applications in rural areas^9^. Consequently, LPG production offers greater economic returns compared to gas condensates or NG^2^.

Process control is a well-established technology that has been widely implemented across various industrial sectors. Over the past few decades, advanced process control has transitioned from laboratory research to standard industrial practice. Many industrial vendors now offer sophisticated solutions, including model predictive control (MPC), which is recognized for its capability to optimize multivariable and constrained processes in an economically efficient manner^10^. MPC encompasses a class of control algorithms that utilize a process model to predict a system’s future response, relying on optimization techniques to determine control actions. At each sampling interval, MPC calculates a sequence of future input signals aimed at optimizing plant performance. However, only the first input in the sequence is applied, while the optimization process is continuously updated at subsequent intervals^11^. In recent decades, MPC has been extensively adopted in the refining and petrochemical industries, demonstrating significant benefits^12,13^. Its implementation enhances product quality, ensures safer operations, and contributes to cost reductions in both energy and raw materials, while minimizing environmental impact^14^. A key element in the effectiveness of MPC strategies is the use of dynamic models, which play a crucial role in predicting and controlling process behavior. Despite the widespread use of MPC, proportional-integral-derivative (PID) controllers have long dominated industrial applications due to their straightforward implementation and tuning procedures. Conventional tuning methods, such as the Ziegler–Nichols approach, have historically provided practical solutions; however, they lack broad applicability and adaptability across diverse process conditions^15^. MPC has been widely established as an advanced strategy for industrial distillation processes, offering predictive capabilities and real-time optimization of control actions. Unlike conventional control approaches, MPC employs a dynamic process model to forecast system behavior over a defined prediction horizon, ensuring continuous optimization of operating conditions^16^. In LPG recovery, MPC plays a critical role in regulating reflux flow and column pressure to improve separation efficiency while minimizing energy consumption^17,18^. Studies have demonstrated that MPC enhances process stability, reduces fluctuations, and maximizes LPG yield by continuously adjusting control inputs based on real-time process data^19^. Despite its advantages, a key limitation of conventional MPC is its reliance on a fixed process model, which may not fully account for variations in feed composition or external disturbances. To address this constraint, AI has been incorporated into MPC frameworks, enabling adaptive learning and real-time model refinement, thereby further enhancing control performance and operational flexibility^20^.

Artificial Intelligence (AI) has transformed process control by enhancing predictive accuracy, optimizing decision-making, and efficiently managing complex multivariable interactions. When integrated with MPC, AI improves control performance by refining model precision, adapting to process variations, and reducing computational complexity^21^. AI techniques, including machine learning and neural networks, enhance MPC by capturing nonlinear relationships and predicting process behavior more effectively than conventional first-principles models^22^. These data-driven approaches enable MPC to account for dynamic process variations and improve disturbance rejection, increasing its robustness under fluctuating operating conditions^23^.

The implementation of AI-driven MPC has yielded substantial benefits in the chemical and petrochemical industries. By training AI models on historical process data, control performance is enhanced, minimizing deviations from setpoints and improving both product quality and process efficiency^24^. Additionally, reinforcement learning techniques have been incorporated into MPC for autonomous tuning and constraint management, reducing reliance on manual controller adjustments and enhancing operational stability^25^. AI-based MPC has also demonstrated significant advantages in optimizing energy consumption by dynamically regulating process variables in response to load variations, resulting in cost reductions and environmental benefits^26^. Research findings indicate that AI-enhanced MPC outperforms conventional approaches in nonlinear systems by improving closed-loop performance, disturbance rejection, and model accuracy^27^. Furthermore, AI enables the development of hybrid MPC frameworks that integrate physics-based and data-driven models. This hybrid methodology enhances predictive capabilities, while preserving physical interpretability, ensuring that AI-generated predictions remain consistent with established process knowledge^28^. Consequently, the integration of AI with MPC has emerged as a promising approach for advancing process control applications, particularly in industries where precision and efficiency are paramount.

To further contextualize this study within emerging trends, several recent innovations in AI-enhanced MPC deserve attention: Transformer-based architectures, such as Transformer MPC, have emerged to accelerate the optimization process within MPC frameworks, enabling faster and more accurate predictions for dynamic systems^29^. Other studies introduced input-augmented Koopman operators, which combine machine learning with linear system theory to effectively model and control complex nonlinear dynamics^30^. Additionally, the integration of input convex neural networks (ICNNs) into MPC has shown promise in improving the computational speed and stability of controllers in nonlinear environments^31^. This capability allows AI-MPC to effectively manage nonlinear process behavior, enhance setpoint tracking, and improve disturbance rejection beyond the capabilities of conventional MPC^32,33^. In LPG recovery applications, AI-driven MPC has demonstrated the ability to reduce control valve fluctuations, stabilize reflux flow, and accelerate convergence to the target recovery percentage^34^. Simulation studies using MATLAB and HYSYS confirm that AI-enhanced MPC improves process stability, lowers energy consumption, and increases separation efficiency. By continuously adapting to real-time process variations, AI-MPC ensures consistent performance even under fluctuating operating conditions^35,36^. These techniques provide new pathways for enhancing control precision, especially in energy-intensive processes such as LPG recovery, where both speed and accuracy are essential.

Although MPC has been widely adopted for improving control performance in industrial processes, its application specifically to LPG recovery units remains limited. Previous studies have mostly focused on steady-state optimization or conventional MPC approaches without systematically investigating dynamic optimization under varying operational disturbances. Moreover, there is a lack of comparative analysis between conventional MPC and AI-enhanced MPC in LPG recovery systems using validated simulation environments. This research addresses this gap by developing and comparing MPC and AI-enhanced MPC strategies to optimize LPG recovery, energy efficiency, and process stability in a debutanizer column.

Recent studies have explored optimization strategies for LPG recovery in debutanizer columns. For instance, one study applied steady-state modeling to improve LPG production^37^. However, this approach was limited to static simulation and did not consider dynamic control strategies such as MPC or AI-enhanced MPC. This underscores the need for an integrated approach that combines steady-state analysis with dynamic process control and intelligent predictive methods. The present study addresses this gap by introducing a methodology that unifies response surface methodology (RSM), dynamic simulation, and AI-enhanced MPC, tailored to fluctuating operating conditions. Unlike conventional methods, this approach handles dynamic disturbances through adaptive control and leverages real plant data, enhancing both responsiveness and industrial applicability.

Several studies have demonstrated the quantitative benefits of AI-enhanced MPC compared to conventional MPC. For instance, applying learning-based MPC in smart building applications resulted in a 40.6% reduction in cooling energy and a 16.7% reduction in heating energy demand^38^. In another case, a stochastic MPC model achieved 7.5% cost savings in HVAC system operation compared to deterministic MPC^39^. A real-world implementation in an institutional building using AI-based MPC resulted in approximately 22% reduction in natural gas consumption and GHG emissions, and a 4.3% reduction in heating demand compared to standard control strategies^40^. These measurable improvements highlight the superior capability of AI-based control approaches to enhance energy efficiency and adapt to varying process conditions more effectively than classical MPC models.

## Methodology

The optimization of LPG recovery from a debutanizer column involves a multidisciplinary approach, integrating process simulation, experimental design, and advanced control strategies. This study is designed to provide a robust framework for analyzing and enhancing the performance of the distillation system under varying operational conditions. Initially, process simulation plays a crucial role by constructing a dynamic model that accurately represents the chemical processes involved, allowing for a thorough investigation of key process variables. The feed molar flow rate, feed pressure, and condenser pressure were selected as the main operating parameters due to their direct influence on phase behavior, separation efficiency, and energy usage in debutanizer columns. These variables were analyzed using RSM under steady-state conditions to evaluate their effects on LPG recovery and determine optimal operating ranges^7,41^. Additionally, these parameters were used as input features for training AI-based models under dynamic conditions to assess their impact on control performance. Previous research has demonstrated the effectiveness of both RSM and AI techniques in process optimization and control^37^. The process simulation was developed based on an actual debutanizer unit from El Wastani Petroleum Company, using operational data to replicate realistic conditions. The design basis includes steady-state operation under typical industrial conditions, with the goal of optimizing LPG recovery. Several assumptions were made to simplify the model, including ideal mixing in the column stages, constant physical properties, and negligible heat loss to the environment. The system was assumed to be isothermal in the dynamic control tests unless otherwise specified. Boundary limits for key variables were initially explored through sensitivity runs in Aspen HYSYS using suggested ranges, and then refined based on observed process responses. Final limits used for RSM were feed molar flow rate from 400 to 520 kgmole/h, feed pressure from 10 to 13 bar, and condenser pressure from 6 to 13.5 bar, as shown in Tables 3 and 9. To systematically assess the influence of these variables, experimental design methods are employed, enabling the identification of optimal operating conditions. In the subsequent phase, process control strategies are introduced, with MPC serving as a fundamental tool to regulate the system and maintain performance stability. Furthermore, the application of AI-enhanced MPC introduces an adaptive control approach, leveraging machine learning algorithms to improve predictive accuracy and system responsiveness. The following sections provide a detailed description of each of these components and their integration within the optimization framework.

### Process simulation and experimental design

In this study, a combination of Aspen HYSYS V14 process simulation and response surface methodology (RSM) is employed to optimize LPG recovery from a debutanizer column. The simulation environment in Aspen HYSYS is configured to model the dynamic behavior of the column, utilizing the Peng-Robinson equation of state (PR EOS) to ensure accurate predictions of phase behavior, thermodynamic properties, and energy balances. The PR EOS is particularly suitable for hydrocarbon systems and non-ideal mixtures, providing reliable process modeling for subsequent optimization analysis.

Following the development of the process model, RSM is applied to systematically investigate the effects of key process variables on LPG recovery. A Box-Behnken Design (BBD) is selected as the experimental design framework due to its efficiency in estimating quadratic effects and capturing factor interactions with a reduced number of simulations runs. This design strategy enables the generation of a statistically sound dataset with minimal computational cost.

Subsequently, statistical analysis is performed using analysis of variance (ANOVA) to assess the significance of individual factors and their interactions. ANOVA results, including p-values, F-values, and coefficients of determination (R^2^), are used to evaluate model adequacy and the predictive capability of the regression model. This analytical approach ensures that the developed empirical model accurately describes the system response and supports further control strategy development. The factor demonstrating the highest statistical significance and practical relevance is identified as the primary disturbance variable for control design. This selection is critical to ensure that the advanced control system is robust against variations in key operating conditions, ultimately enhancing LPG recovery performance under dynamic scenarios.

### Advanced process control in LPG recovery optimization

Efficient process control is essential for optimizing LPG recovery units in NGLs plants. The separation of LPG components, primarily propane and butane, depends on precise regulation of reflux flow rate, column pressure, and product purity. Given the dynamic nature of distillation processes and the variability in feed composition and operating conditions, advanced control strategies are required to manage multivariable interactions and enhance operational stability.

MPC has gained prominence as a reliable approach due to its predictive capabilities and ability to enforce operational constraints. Unlike traditional control methods, MPC continuously anticipates process behavior and adjusts control actions, accordingly, ensuring stable and efficient operation. Recently, the integration of AI into MPC frameworks has further improved adaptability and robustness. AI-enhanced MPC leverages machine learning algorithms to refine model predictions and optimize control performance under varying process conditions. This section examines the role of MPC in optimizing LPG recovery and discusses the advantages of AI-enhanced MPC in improving control efficiency, reducing energy consumption, and enhancing overall system performance stability.

#### Strategic framework of MPC for process optimization

Efficient process control is essential for optimizing LPG recovery units in NGLs plants. The separation of LPG components, primarily propane and butane, depends on precise regulation of reflux flow rate, column pressure, and product purity. Given the dynamic nature of distillation processes and the variability in feed composition and operating conditions, advanced control strategies are required to manage multivariable interactions and enhance operational stability.

**Fig. 1:**
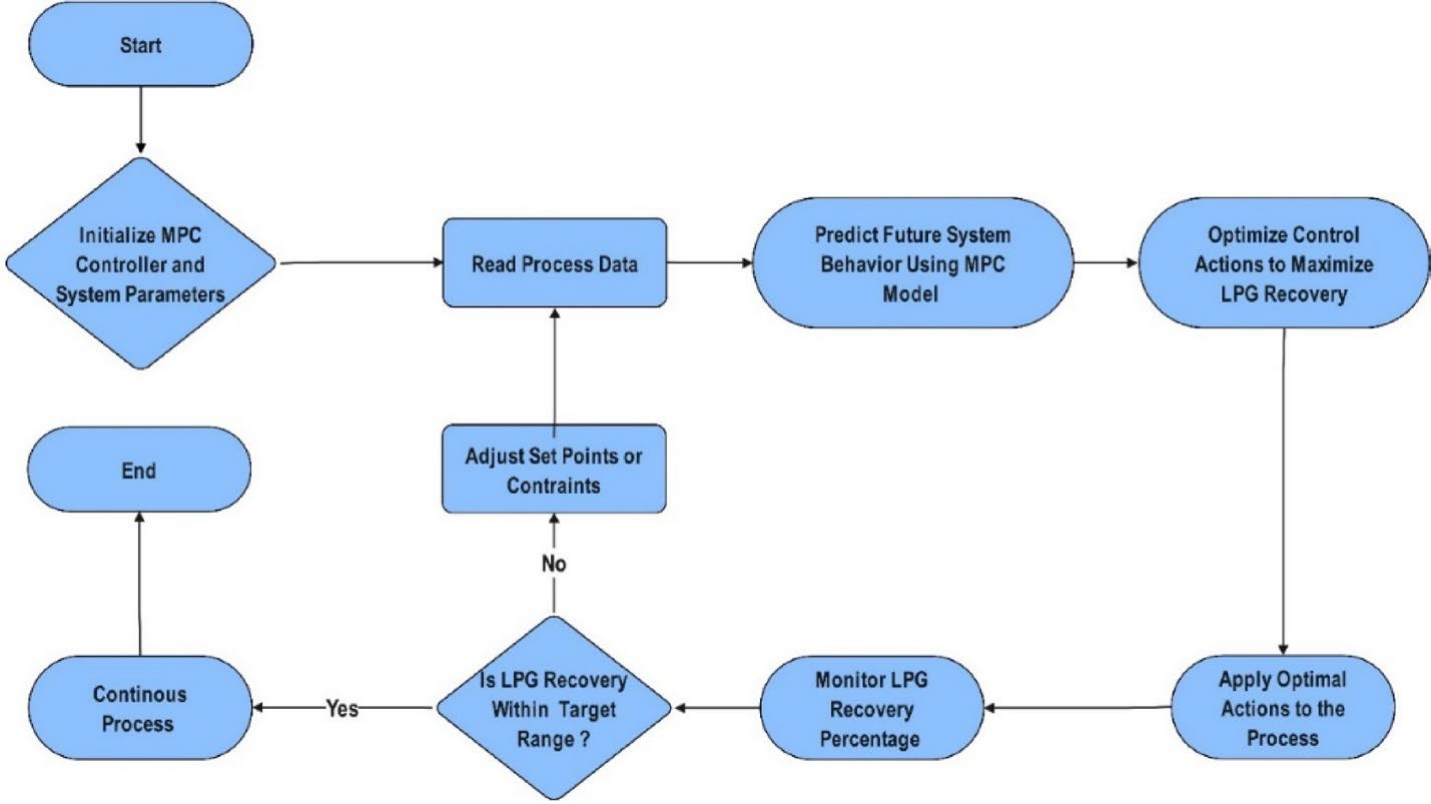
Process flow of MPC algorithm.

MPC has gained prominence as a reliable approach due to its predictive capabilities and ability to enforce operational constraints. Unlike traditional control methods, MPC continuously anticipates process behavior and adjusts control actions, accordingly, ensuring stable and efficient operation. Figure 1 outlines the structure of the MPC system, designed to optimize process performance through predictive modeling. The controller continuously reads process data, forecasts system behavior, and fine-tunes setpoints to maintain optimal LPG recovery. By leveraging a mathematical model, the MPC ensures stable operation while responding to process variations.

#### AI-enhanced MPC: a machine learning approach for adaptive control

**Fig. 2:**
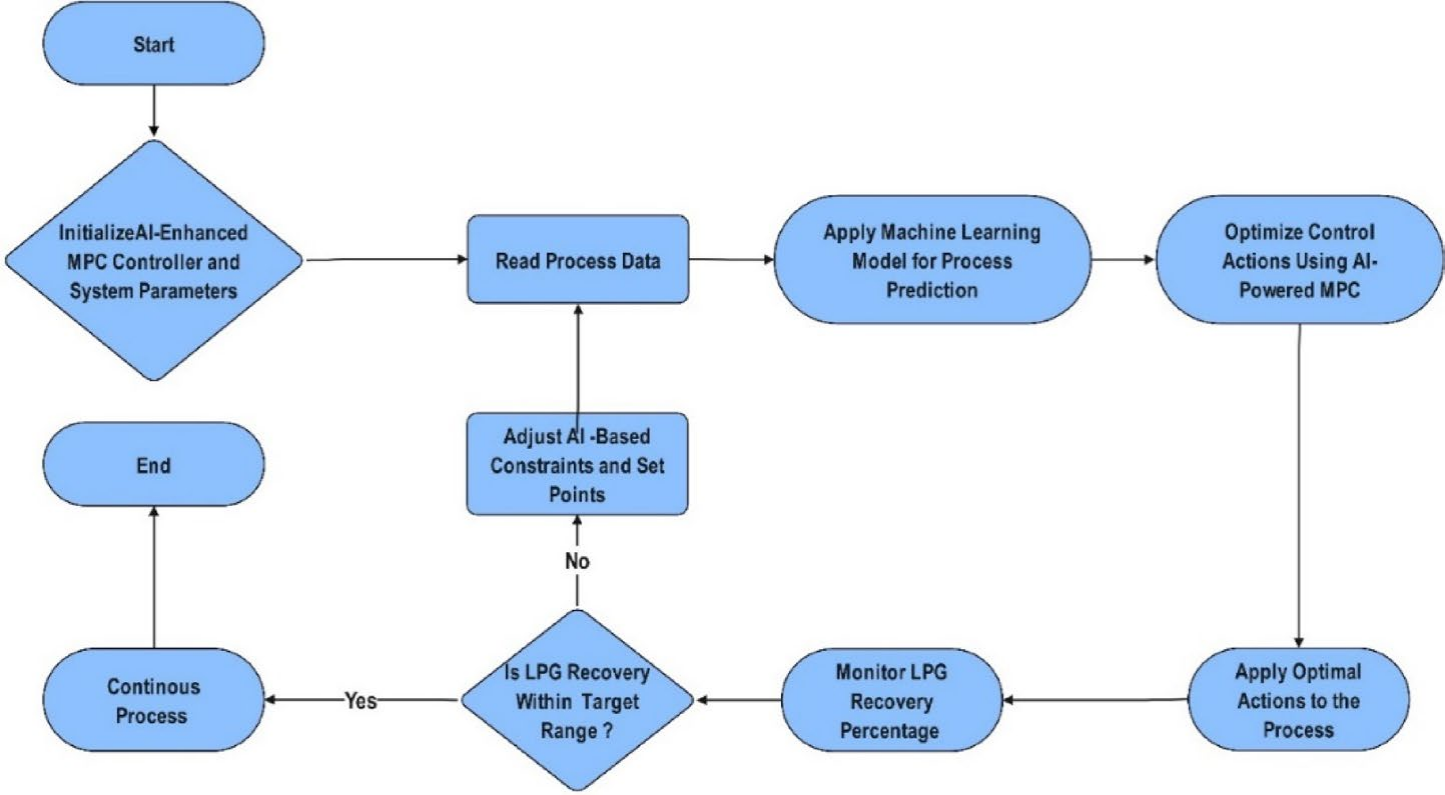
Process flow of AI-enhanced MPC algorithm.

The integration of AI with MPC represents a major advancement in process automation, enabling adaptive and self-optimizing control strategies. AI-enhanced MPC leverages machine learning techniques, including artificial neural networks (ANNs) and decision trees, to analyze historical process data and dynamically adjust control parameters in real time. Figure 2 presents a generalized AI-enhanced MPC framework that illustrates the core components of the methodology implemented in this study. While the diagram outlines a system-level control structure, the actual implementation applied this framework using process-specific inputs is clarified in case study section. Instead of relying solely on predefined models, this system dynamically adapts based on AI-driven predictions, refining control actions in real time. By continuously optimizing constraints and decision-making, AI-powered MPC enhances accuracy, responsiveness, and overall process efficiency. These findings underscore the potential of AI-driven control methodologies in industrial applications, positioning AI-MPC as a more efficient, adaptive, and sustainable approach to optimizing LPG recovery. The key performance indicators (KPIs) targeted in this study include LPG recovery percentage, reboiler and condenser duties, reflux flow rate, and settling time. While temperature and pressure were monitored throughout the study to assess system stability, they were not used as primary performance indicators for model comparison.

## Case study

### Process simulation

**Fig. 3:**
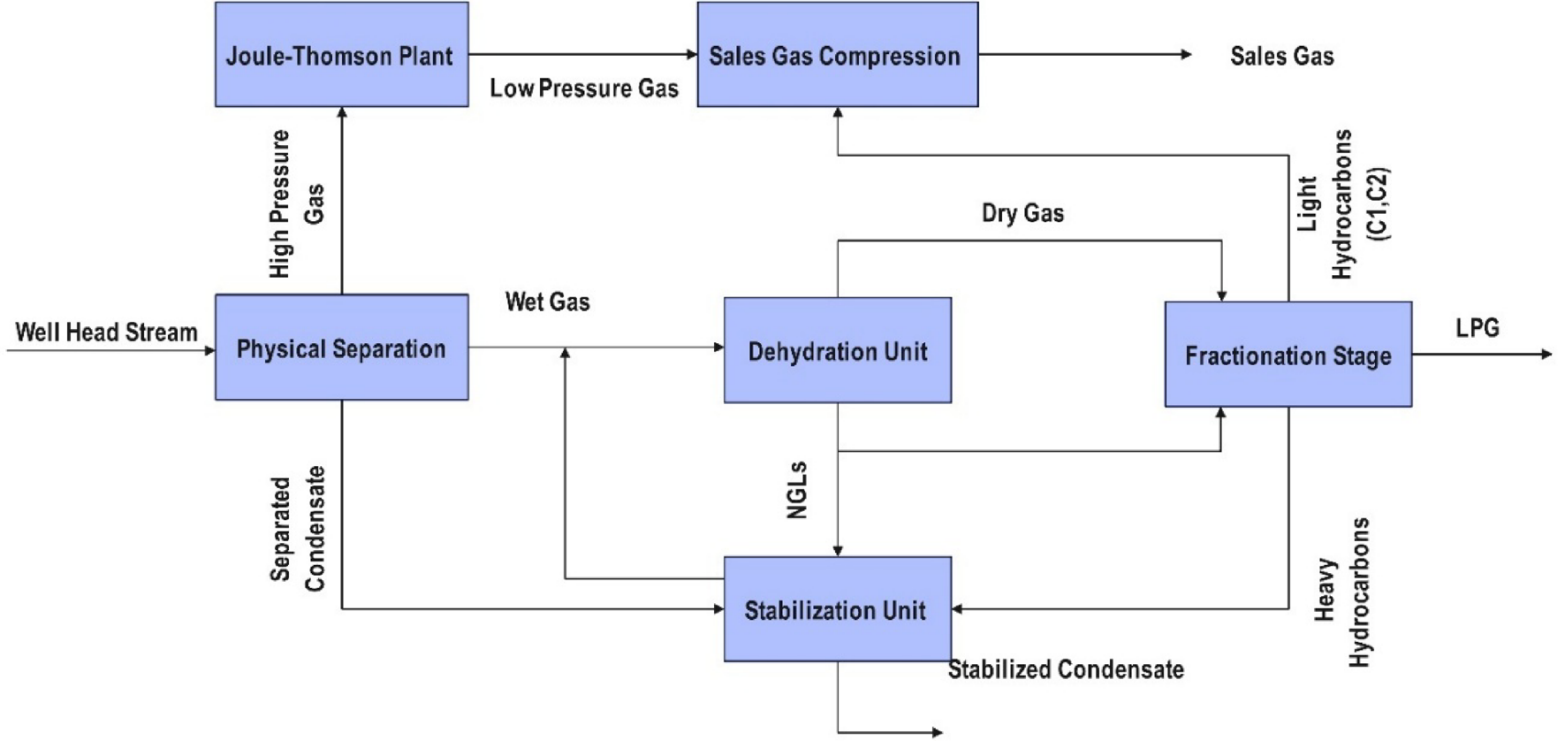
Block diagram for NGLs plant.

This case study investigates an NGL fractionation system; the process configuration and data are adopted from a previously published model^42^, which was developed using actual plant data from El Wastani Petroleum Company. The selected case study reflects an updated version of the original process, offering a reliable basis for evaluating control strategies under realistic operating conditions. The process begins with physical separation, where the wellhead stream enters a separator, resulting in the separation of gas, condensate, and water. The gas stream is directed to the Joule-Thomson (JT) plant, where it undergoes cooling and expansion to facilitate the condensation of heavier hydrocarbons. The remaining gas is subsequently transferred to the sales gas compression unit, where it is compressed and delivered as sales gas. Simultaneously, the liquid phase from the physical separation stage is sent to the dehydration unit, where water is removed to prevent hydrate formation. The dehydrated hydrocarbon stream is then processed in the fractionation stage, where various hydrocarbon fractions are separated. This step facilitates the recovery of LPG, including propane and butanes, while directing other components for further processing. Additionally, certain liquid hydrocarbons undergo stabilization in the Stabilization Unit, where lighter components are removed, yielding stabilized condensate as the final product. The overall process is illustrated in Fig. 3.

**Table 1:** Feed specifications of the NGLs plant^42^.

Feed	Raw gas	Raw condensate
Pressure (bar)	50.31	50.31
Temperature (°C)	38	38
Molar flow rate (kgmole/h)	8044	170.8
Mole fractions		
N_2_	0.0004	0.0
CO_2_	0.0032	0.0005
C_1_	0.8699	0.0372
C_2_	0.0746	0.0229
C_3_	0.0272	0.0209
i-C_4_	0.0081	0.0257
n-C_4_	0.0062	0.0346
i-C_5_	0.0030	0.0512
n-C_5_	0.0017	0.0371
C_6_	0.0019	0.1047
C_7_	0.0013	0.1621
C_8_	0.0006	0.1972
C_9_	0.0001	0.1131
C_10_	0.0	0.0680
C_11_	0.0	0.0383
C_12_	0.0	0.0257
C_13_	0.0	0.1092
C_14_	0.0	0.0127
C_15_	0.0	0.0116
C_16_	0.0	0.0058
C_17_	0.0	0.0056
C_18_	0.0	0.0030
C_19_	0.0	0.0010
C_20_	0.0	0.0007
C_21_	0.0	0.0005
C_22_	0.0	0.0003
C_23_	0.0	0.0004
H_2_O	0.0018	0.0

**Table 2:** Feed specifications of the debutanizer unit.

Parameter	Value
Column configuration	
Number of sieve trays	24
Feed location	Tray 10
Column pressures (bar)	12.39–12.73
Total condensation	Yes
Reflux molar flow (kgmole/h)	281.2
Boil up molar flow (kgmole/h)	272.5
Feed properties	
Molar flow rate (kgmole/h)	493.5
Temperature (°C)	97.72
Pressure (bar)	13
Vapor fraction	0.6132
Feed composition (mole fractions)	
C_1_	0.0
C_2_	0.0
C_3_	0.4511
i-C_4_	0.1411
n-C_4_	0.1133
i-C_5_	0.0669
n-C_5_	0.0407
C_6_	0.0674
C_7_	0.0744
C_8_	0.0330
C_9_	0.0083
C_10_	0.0026
C_11_	0.0008
C_12_	0.0003
C_13_	0.0001

**Fig. 4:**
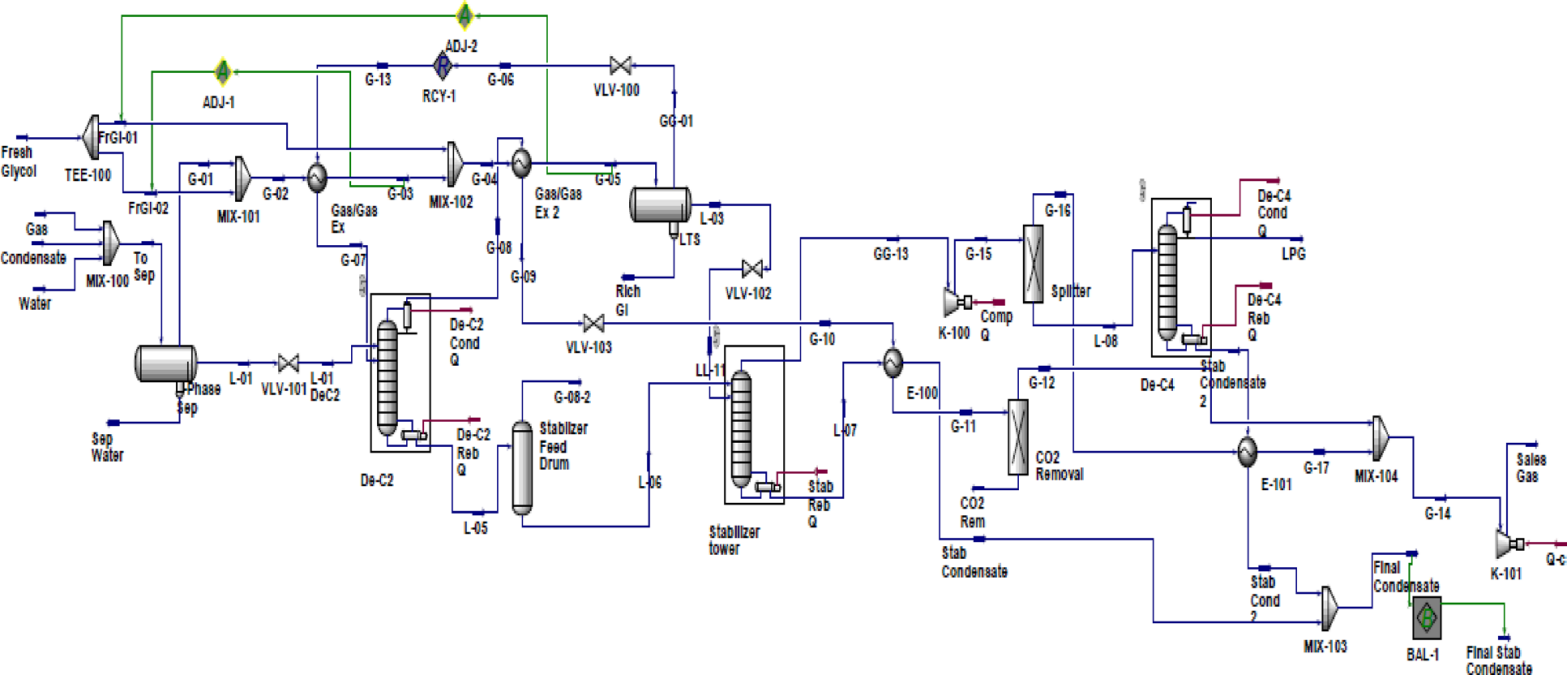
Simulation of the NGLs plant^42^.

The process simulation of the design case study integrates an optimized model between the dehydration unit and the NGLs recovery unit, specifically the heavy component removal section of the LPG plant. This integration achieves the target production of sales gas while maximizing the recovery of propane, normal butane, and iso-butane (LPG), as well as stabilized condensate (C5^+^)^42^. Before entering the low-temperature processing unit, the NG feed must undergo purification to remove condensate (C5^+^) and water, ensuring the gas meets sale or disposal requirements^43^. The LPG plant consists of two primary sections: the dehydration unit and the NGL recovery (Heavy Component Removal) section, as illustrated in Fig. 3. The dehydrated gas is then processed in the NGL recovery unit to remove heavy components and is subsequently delivered as sales gas to the Egyptian National Gas Grid, adhering to specified quality standards. The NGL plant design aims to enhance the economic value of the feed gas stream by incorporating a restructured LPG recovery unit that utilizes a low-temperature separation technique to recover LPG through an integrated process^42^. The low-temperature separation (LTS) unit, also referred to as straight refrigeration or low-temperature extraction (LTX), is employed for dew point control and gas conditioning. This process involves cooling and partial condensation of the gas stream. When the inlet pressure is sufficiently high to meet discharge pressure requirements while maintaining an acceptable pressure drop, cooling is achieved via expansion through a Joule-Thomson (J-T) valve. Otherwise, external refrigeration is required, modified by incorporating heat recovery through inter-exchange mechanisms^44^. Hydrate formation is a critical issue in pipeline transportation of water-saturated NG. To mitigate this risk, chemical drying agents are introduced, with ethylene glycol (EG) being the most commonly used inhibitor^45^. The feed stream, consisting of three phases, initially undergoes separation in a three-phase separator, which segregates the gas, water, and condensate. Gas bubbles rise, removing free water and separating condensate. The gas is then cooled in two heat exchangers arranged in series (Gas/Gas Ex and Gas/Gas Ex-2). To prevent hydrate formation, an 80%−20% ethylene glycol/water mixture is directly injected at a total flow rate of 35.928 m³/day. This mixture is distributed into the Gas/Gas Ex at 34.344 m³/day and into Gas/Gas Ex-2 at 1.584 m³/day upstream of each heat exchanger to absorb any entrained water in the gas stream. The gas stream is then directed to a three-phase low-temperature separator (PH-III LTS), where any remaining water droplets and glycol-rich solution are removed from the condensate. The stream undergoes additional cooling via a J-T valve, facilitating refrigeration. Heat recovery is achieved through two streams: the deethanizer column overhead stream (G-08), which cools Gas/Gas Ex-2, and the Gas/Gas Ex exchanger itself. The processed gas subsequently passes through a CO₂ removal bed to capture carbon dioxide, ensuring compliance with sales gas specifications. Following dehydration, the LPG recovery unit is introduced to remove all remaining condensate and water from the gas^46^. The separation process is restructured within fractionation towers, which include the deethanizer, debutanizer, and stabilizer columns. The optimized, integrated process model is depicted in Fig. 4.

The deethanizer receives two feed streams: a liquid stream (L-01) from the three-phase separator, which is injected at tray 14, and a gas stream (G-07) from the PH-III LTS separator, injected at tray 12. The feed location is chosen based on the tower composition, which should closely match the feed composition to ensure efficient separation. The deethanizer column is designed with 28 trays. The condenser operates at a pressure of 29.75 bar under full reflux conditions, while the reboiler operates at 29.92 bar. This column separates methane and ethane, along with traces of carbon dioxide, from the heavier hydrocarbons. The overhead stream contains light hydrocarbons, while the bottom stream consists of C3^+^ components.

The deethanizer bottom stream is directed to the stabilizer feed drum, where it serves as the primary feed for the condensate stabilizer column. The first feed stream (L-06) is injected at the top tray, while a second liquid stream (L-11) from the PH-III LTS separator is injected at tray 12. The stabilizer column is designed with 14 trays and functions to reduce the vapor pressure of the condensate by removing lighter components. This process is facilitated by the reboiler, where lighter components ascend as vapor through the overhead gas stream (GG-13), while the heavier fraction accumulates at the bottom is cooled using inter-exchange heat recovery (E-104). A portion of the liquid is recirculated through the reboiler to maintain the column’s thermal balance.

The overhead gas stream (GG-13) from the stabilizer column is compressed in K-104 to 13.5 bar before entering the splitter, where lighter hydrocarbons (C_1_ and C_2_) are separated as stream G-16, while heavier components (C3^+^) form stream L-08. The L-08 stream serves as the debutanizer feed, entering at tray 10. The debutanizer column facilitates the separation of light distillates (C_3_, n-C_4_, i-C_4_) from heavier hydrocarbons. LPG is recovered through total condensation of the overhead distillate, while light naphtha is separated as the bottom product^2^.

The NGLs plant’s feed specifications are summarized in Table 1, while the debutanizer unit’s specifications, which are the primary focus of this study, are presented in Table 2. The debutanizer column is designed with 24 trays. The feed stream (LL-10) is introduced at tray 10, with the condenser operating at 12.39 bar and the reboiler at 12.73 bar. According to the published reference model^42^ from which the process configuration and operating data were adopted, pressure drops across the debutanizer and associated equipment were assumed to be zero (ΔP = 0). This assumption enabled a smooth and consistent transition from steady-state simulation to dynamic simulation, which is essential for MPC implementation. Since the study focuses on evaluating dynamic control performance rather than hydraulic design, this simplification does not compromise the validity of the results.

### Application of RSM for LPG recovery enhancement: key input variables

In this case study, three critical operating variables are selected for optimizing LPG recovery from the debutanizer column. These include feed molar flow rate, feed pressure, and condenser pressure. The selection is based on engineering judgment, process relevance, and preliminary steady-state simulations, in addition to practical considerations regarding which inputs can be modified within the Aspen HYSYS simulation environment. These parameters represent the most direct and controllable influences on LPG recovery. Feed composition was treated as a fixed input in this study to minimize confounding effects and reflect stable upstream supply conditions. While composition influences recovery, it is typically not a directly controllable variable in real-time operation, unlike flow rate and pressure.

To systematically investigate their effects and potential interactions, the Box-Behnken Design (BBD) is employed. This statistical design efficiently models quadratic responses while minimizing the number of required simulations runs. For the selected three factors, the BBD generates a total of 17 experimental runs, covering all combinations of high, medium, and low levels. The defined ranges and coded levels for each variable reflect realistic and feasible operating conditions for the debutanizer column. The selected variables and their corresponding levels are presented in Table 3.

**Table 3:** Operating parameters and bounds assumptions for RSM optimization.

Factor	Units	Level		
		Low (−1)	Medium (0)	High (+ 1)
Feed molar flow rate (A)	kgmole/h	400	460	520
Feed pressure (B)	Bar	10	11.5	13
Condenser pressure (C)	Bar	6	9.75	13.5

### MPC implementation in debutanizer column

The MPC controller is implemented with predefined parameters, including a step response length of 100, a prediction horizon of 30 steps, and a control horizon of 5 steps. The controller operates at a 30-second control interval, which is shorter than the system’s time constant of 40 s. The time constant represents the duration it takes for the LPG recovery percentage to reach 63.2% of its final value following a change in the process. By setting the control interval to 30 s, which is lower than the time constant, the MPC can make frequent adjustments and respond effectively to changes in the system, ensuring continuous stabilization of LPG recovery. The manipulated variable (reflux flow rate), represented by the control valve opening (0%−100%), is regulated to maintain the LPG recovery percentage within the range of 99.73–99.85%. The shorter control interval allows the MPC to adjust in real-time, optimizing the recovery process while accommodating the slower dynamics of the debutanizer column. Additionally, the prediction horizon is set to 30 steps (equivalent to 15 min of process foresight), ensuring the controller can anticipate process variations and take proactive actions. This setup allows the MPC to stabilize the recovery percentage and enhance it beyond the steady-state setpoint, demonstrating its ability to optimize column performance and improve overall process efficiency. The input data to the MPC controller is presented in Table 4.

**Table 4:** Key assumptions and setup parameters for MPC design.

Parameter	Value
Step response length	100
Prediction horizon	30
Control horizon	5
Control interval (seconds)	30
Process variable (min-max)	99%−100%
Manipulated variable(min-max)	250–320 kgmole/h
Set point (min-max)	99.73–99.85%

### Application of regression tree for predicting LPG recovery in AI-enhanced MPC control system

This section explores the use of a regression tree model for predicting LPG recovery percentage, based on key control variables such as control valve opening and setpoint. A Decision Tree, specifically a regression tree (using fitrtree), is a form of supervised learning model that predicts continuous values. The model is integrated into an enhanced MPC framework to optimize the control strategy, improving dynamic decision-making and the accuracy of recovery predictions. Table 5 presents the specification of the regression tree model integrated into the AI-enhanced MPC framework, including input variables, model type, and integration method. In addition to the regression tree, an artificial neural network (ANN) was also trained using the same input features to enhance the accuracy of recovery prediction. While the regression tree offered high interpretability and fast execution, the ANN model captured complex nonlinear interactions between the control inputs and LPG recovery. Both models were integrated into the AI-enhanced MPC structure to support adaptive control and improve the robustness of the system under variable conditions. The regression tree provided quick decision-making, while the ANN contributed to enhancing prediction robustness and adaptability in real-time, enabling the controller to respond more effectively to dynamic disturbances and optimize recovery under changing operating conditions.

**Table 5:** Model specification for regression tree in AI-enhanced MPC control system.

Item	Description
Algorithm used	Regression tree (using fitrtree)
Inputs	Control valve opening (%)
	Setpoint (LPG recovery %)
	Time
	Predicted LPG recovery from MPC
Outputs	Final predicted LPG recovery percentage (AI-enhanced MPC)
Model type	Supervised learning regression tree
Integration with control	The AI model refines the predicted LPG recovery generated by the MPC controller by analyzing the relationship between control actions and recovery performance. This enables adaptive and intelligent decision-making, enhancing process stability and recovery efficiency
Stable points selection	Data was trimmed to include only stable regions, where process variables such as control valve opening exhibited minimal fluctuations, ensuring accurate learning
Model output	Comparison of actual vs. AI-enhanced MPC predictions, demonstrating improved accuracy and control precision within the LPG recovery target range

## Results and discussions

### Steady-state process simulation

**Fig. 5:**
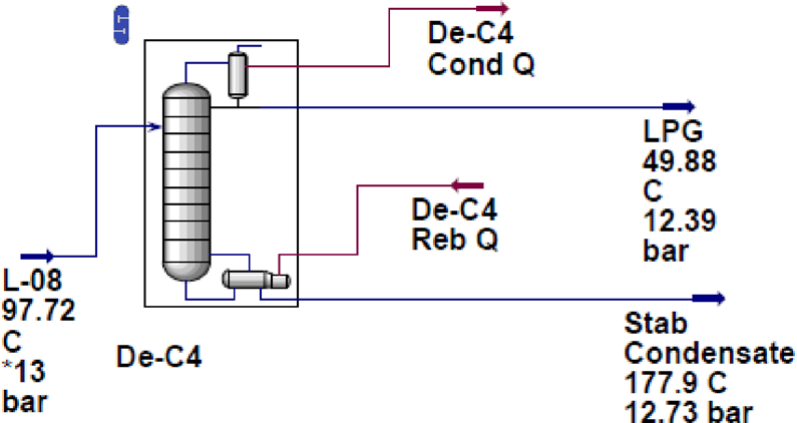
Simulation of debutanizer unit at steady-state.

The steady-state simulation of the debutanizer unit, as illustrated in Fig. 5, established a baseline for assessing operational performance. Key process variables, including temperature, pressure, and composition profiles, were analyzed to determine the optimal conditions required for efficient separation. The results provided valuable insights into the distribution of components along the column, particularly the effective separation of butane from heavier hydrocarbons. These findings served as a critical reference for dynamic simulations, highlighting the importance of maintaining specific temperature and pressure gradients to ensure compliance with product specifications and enhance overall separation efficiency.

The analysis revealed key operational constraints, including high reboiler duty and condenser load, which were effectively managed through dynamic control strategies. The system successfully achieved an LPG recovery of 99.73%, with a molar flow rate of 360.4 kgmole/h. The C_3_ recovery reached 100%, while i-C_4_ and n-C_4_ recovery percentages were 99.87% and 98.48%, respectively. The condenser and reboiler duties were recorded at 2,493,000 kcal/h and 1,557,000 kcal/h, ensuring efficient heat exchange and energy utilization. The reflux molar flow was maintained at 281.2 kgmole/h, contributing to stable separation performance. Additionally, the temperature and pressure profiles of LPG remained steady at 49.88 °C and 12.39 bar, confirming optimal operating conditions. These results demonstrate the effectiveness of the applied control strategies in maintaining process stability while maximizing LPG recovery efficiency.

### Detailed analysis of LPG recovery factors

The response variable reported in Table 6 was measured as the molar flow rate of LPG in the overhead product, pressure of the feed, and condenser pressure change. These were derived from simulation runs in Aspen HYSYS and guided by a BBD.

**Table 6:** Simulated run design derived using BBD.

Run	A (kgmole/h)	B (bar)	C (bar)	Simulated recovery (kgmole/h)	Predicted recovery (kgmole/h)	Residual
1	460	13	13.5	336.2	336.16	0.0375
2	460	11.5	9.75	333.9	333.82	0.0800
3	520	13	9.75	379.1	379.13	−0.0250
4	460	10	6	332.2	332.24	−0.0375
5	520	10	9.75	375.7	375.70	0.0000
6	400	10	9.75	288.9	288.88	0.0250
7	400	11.5	13.5	290.9	290.94	−0.0375
8	460	11.5	9.75	333.8	333.82	−0.0200
9	400	11.5	6	290.2	290.19	0.0125
10	520	11.5	13.5	378.2	378.21	−0.0125
11	460	13	6	335.3	335.31	−0.0125
12	460	11.5	9.75	333.8	333.82	−0.0200
13	520	11.5	6	377.3	377.26	0.0375
14	400	13	9.75	291.6	291.60	0.0000
15	460	10	13.5	333.1	333.09	0.0125
16	460	11.5	9.75	333.8	333.82	−0.0200
17	460	11.5	9.75	333.8	333.82	−0.0200

### Fit summary and model selection

The fit summary, presented in Table 7, guided the selection of the quadratic model as the most appropriate, with the following metrics:

**Table 7:** Comparison between models fit to LPG recovery data.

Source	Sequential *p*-value	Lack of fit *p*-value	Adjusted *R*^2^	Predicted *R*^2^	
Linear	< 0.0001	0.0017	0.9999	0.9999	
2FI	0.5185	0.0013	0.9999	0.9999	
**Quadratic**	**< 0.0001**	**0.4028**	**1.0000**	**1.0000**	**Suggested**
Cubic	0.4028		1.0000		**Aliased**

**Fig. 6:**
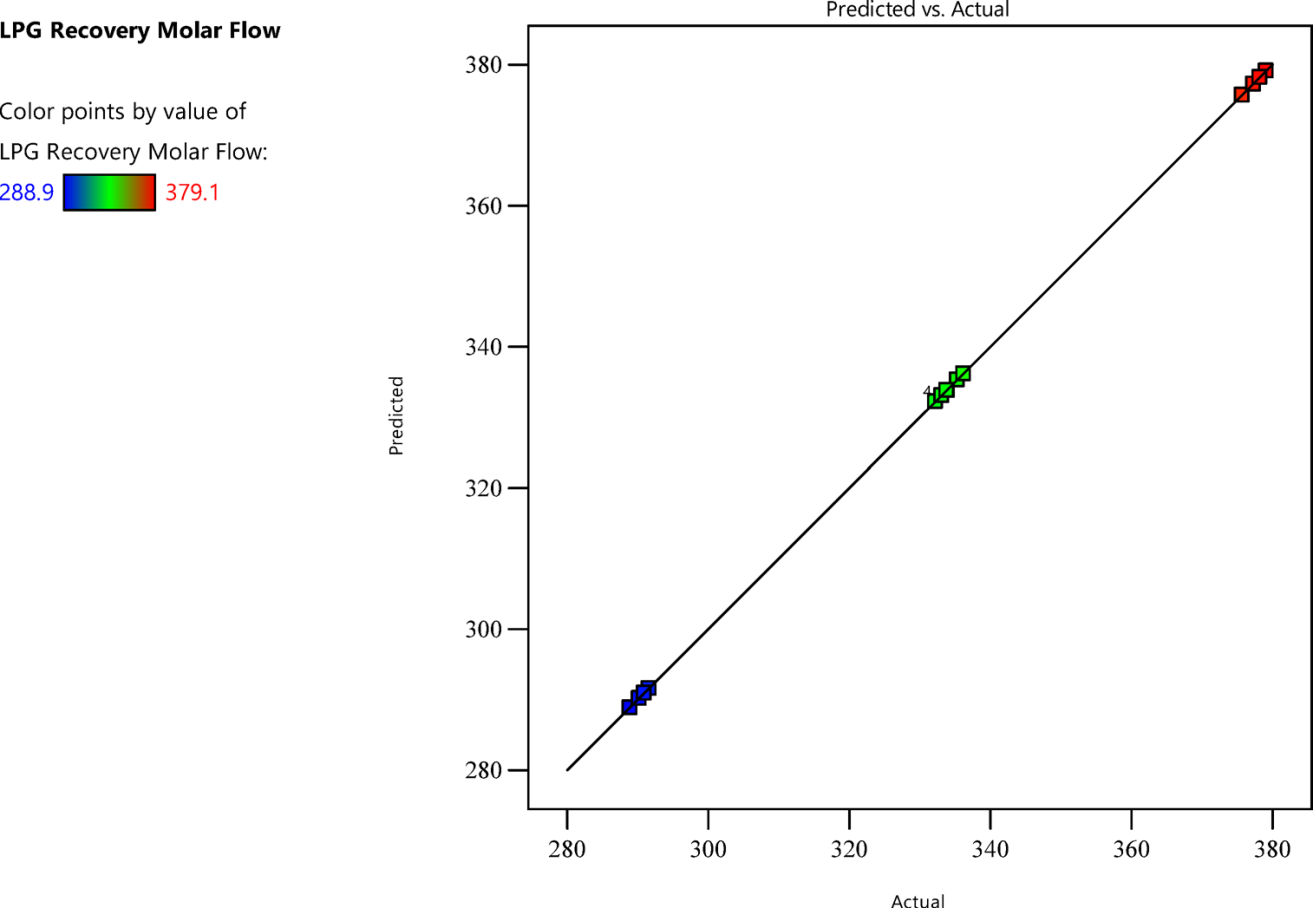
Comparison between predicted and actual data form process simulator.

The quadratic model was selected due to its low sequential p-value and non-significant lack of fit (p-value = 0.4028), indicating good fit without overfitting. Figure 6 shows the relationship between the HYSYS-based simulation data and the quadratic model-based LPG recovery data. The adjusted R^2^ of 0.999998 and predicted R^2^ 0.999991 suggest the model captures nearly all variability, an unexpected detail given the complexity of process simulations. The proposed model equation in coded factors can be written as:

1 $$\:LPG\:Recovery=333.82+43.5875\:\varvec{A}+1.5375\:\varvec{B}+0.425\:\varvec{C}+0.175\:\varvec{A}\varvec{B}+0.05\:\varvec{A}\varvec{C}-0.0225\:{\varvec{A}}^{2}+0.0275\:{\varvec{B}}^{2}+0.3525\:{\varvec{C}}^{2}$$

### ANOVA for quadratic model

ANOVA results, shown in Table 8, confirm the model’s significance with an F-value of 763726.70 and p-value < 0.0001, implying less than 0.01% chance of occurring due to noise. Significant model terms (p-value < 0.05) are shown in the ANOVA table. The lack of fit F-value of 1.25 (p-value = 0.4028) is not significant, indicating the model adequately fits the data relative to pure error, which is desirable.

**Table 8:** ANOVA analysis for the simulated LPG recovery molar flow rates.

Source	Sum of squares	df	Mean square	F-value	*p*-value	
**Model**	15219.98	9	1691.11	7.637E + 05	< 0.0001	Significant
A-Feed molar flow	15198.96	1	15198.96	6.864E + 06	< 0.0001	
B-Feed pressure	18.91	1	18.91	8540.56	< 0.0001	
C-Condenser pressure	1.44	1	1.44	652.58	< 0.0001	
AB	0.1225	1	0.1225	55.32	0.0001	
AC	0.0100	1	0.0100	4.52	0.0712	
BC	0.0000	1	0.0000	0.0000	1.0000	
A^2^	0.0021	1	0.0021	0.9626	0.3592	
B^2^	0.0032	1	0.0032	1.44	0.2695	
C^2^	0.5232	1	0.5232	236.28	< 0.0001	
**Residual**	0.0155	7	0.0022			
Lack of fit	0.0075	3	0.0025	1.25	0.4028	Not significant
Pure error	0.0080	4	0.0020			
**Cor total**	15220.00	16				

The results indicate that feed molar flow rate (A) has the most significant positive linear effect on LPG recovery, with a coefficient of 43.5875 and a p-value of < 0.0001, suggesting that increasing feed flow rate substantially increases LPG recovery. This is expected in process engineering, as higher feed flow rates can enhance separation efficiency, provided the column does not flood. Feed pressure (B) and condenser pressure (C) have smaller positive linear effects, with coefficients of 1.5375 and 0.425, respectively, and p-values indicating significance. The interaction between feeding molar flow and feed pressure (AB), with a coefficient of 0.175 and p-value of 0.000145, is significant, implying that their combined effect on LPG recovery is greater than the sum of their individual effects. This interaction suggests that the impact of feed flow on recovery varies with feed pressure, potentially due to changes in column hydraulics. The quadratic term of condenser pressure (C^2^), with a coefficient of 0.3525 and p-value of < 0.0001, is significant, indicating a non-linear relationship. This suggests that condenser pressure has an optimal range for maximizing LPG recovery, beyond which further increases may reduce efficiency due to altered vapor-liquid equilibrium. Non-significant terms, such as AC (p-value = 0.071), A^2^ (p-value = 0.359), and B^2^ (p-value = 0.269), were retained for hierarchy but do not contribute substantially to the model. The lack of fit being non-significant (p-value = 0.4028) confirms the model’s adequacy.

**Fig. 7:**
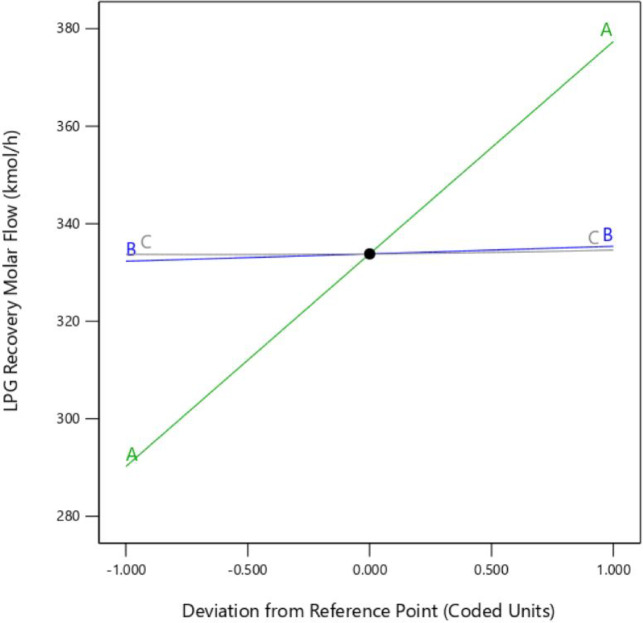
Perturbation plot showing the effect of feed molar flow rate (A), feed pressure (B), and condenser pressure (C) on LPG recovery, with A having the strongest influence.

The perturbation plot as shown as in Fig. 7 visually reinforces that feed molar flow rate (factor A) is the dominant factor influencing LPG recovery, with a clear upward slope indicating its substantial positive effect. This aligns with the ANOVA results, where factor A had the largest F-value (6864047.02) and lowest p-value < 0.0001, confirming its statistical significance. For feed pressure (factor B) and condenser pressure (factor C), the plot’s near-horizontal lines suggest minimal visual impact, which is an unexpected detail given their statistical significance in the model (p-values of 4.58 × 10^−12^ and 3.60 × 10^−8^, respectively). However, the small changes (3.075 kgmole/h for B and 0.85 kgmole/h for C) are consistent with their smaller coefficients (1.5375 and 0.425), and their effects are less pronounced on the plot’s scale (y-axis range 280–380 kgmole/h). The interaction term AB (p-value = 0.000145) and quadratic term C^2^ (p-value = 1.19 × 10^−6^) are significant, but their effects are not directly visible in the perturbation plot, which primarily shows linear deviations. This plot is thus a simplified visualization, focusing on main effects, and complements the detailed statistical analysis.

### Analysis of operating parameters

This section analyzes how operating parameters, feed molar flow rate, feed pressure, and condenser pressure, affect LPG recovery in a debutanizer column, using RSM plots. RSM helps visualize the relationships and interactions between these parameters and the response variable, LPG recovery molar flow, providing insights for optimizing process control. The following sections discuss the effect of each parameter on LPG recovery, supported by RSM plots that illustrate the main effects and interactions.

#### Effect of feed molar flow rate on the response variable

**Fig. 8:**
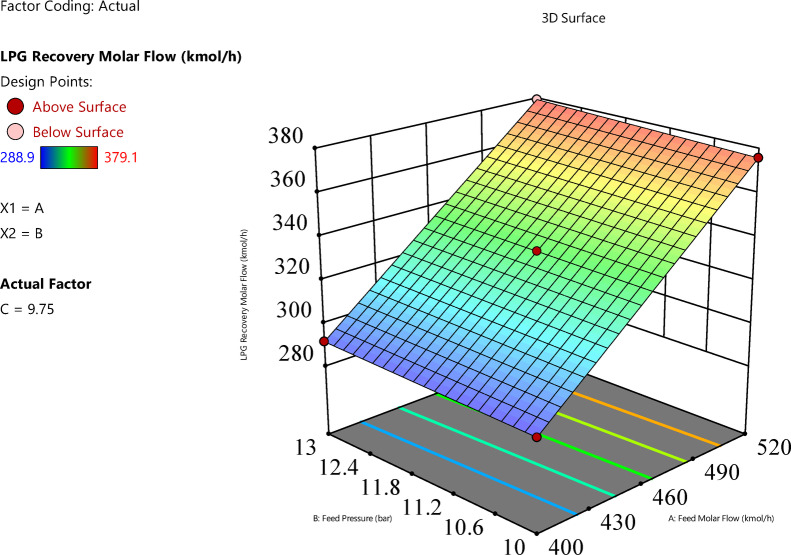
3D representation of the interaction effect between feed molar flow rate and feed pressure on LPG molar flow rate.

**Fig. 9:**
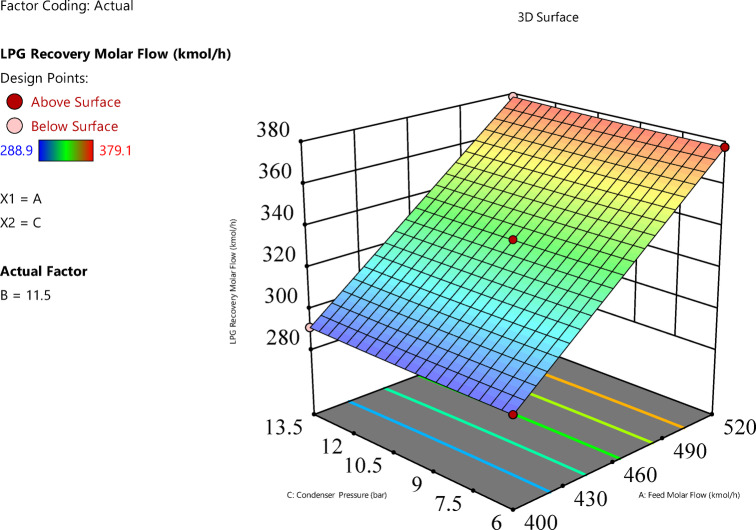
3D representation of the Interaction effect between feed molar flow rate and condenser pressure on LPG molar flow rate.

Feed molar flow rate significantly increases LPG recovery, with a positive linear effect (p-value < 0.0001). Figure 8 (feed molar flow rate vs. feed pressure) shows that as the feed molar flow rate increases from 400 to 520 kgmole/h, LPG recovery rises from approximately 280 to 380 kgmole/h at constant feed pressure. The interaction with feed pressure (p-value = 0.000145) indicates that higher feed pressures amplify the positive effect of molar flow rate, resulting in greater recovery when both variables are high. Figure 9 (feed molar flow rate vs. condenser pressure) confirms this trend, showing increased recovery with higher feed molar flow rates across condenser pressure levels, though the interaction with condenser pressure is less significant (p-value = 0.071).

#### Analysis of pressure effects

1. Effect of feed pressure on the response variable.
  Feed pressure has a significant positive linear effect on LPG recovery (p-value < 0.0001), with a coefficient of 1.5375, indicating a smaller but notable impact. This impact is slight and hard to notice, which is consistent with the effect plot shown in Fig. 10. Figure 11b illustrates this effect, showing that at a constant feed molar flow rate, increasing feed pressure from 10 to 13 bar results in an increase in LPG recovery. For example, at a feed molar flow rate of 460 kgmole/h, recovery increases from approximately 332.2 to 335.3 kgmole/h as feed pressure increases. Based on the plot’s surface and color gradient in Fig. 10, The significant interaction term AB (coefficient 0.175, p-value = 0.000145) suggests that the effect of feed pressure on LPG recovery is enhanced at higher feed molar flow rates, as seen in the steeper response surface at higher feed molar flow rates.
  **Fig. 10:**
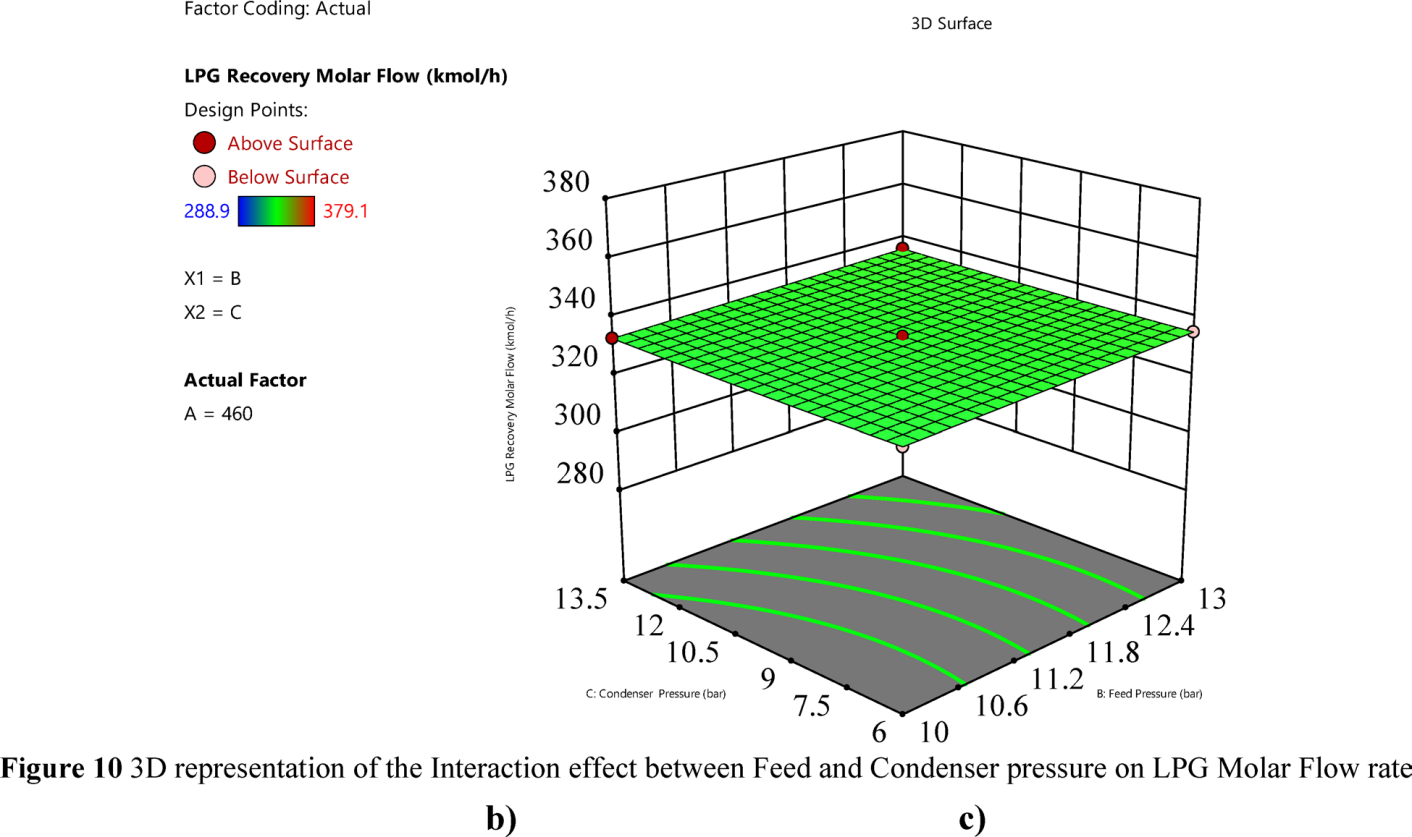
3D representation of the Interaction effect between Feed and Condenser pressure on LPG Molar Flow rate.
  **Fig. 11:**
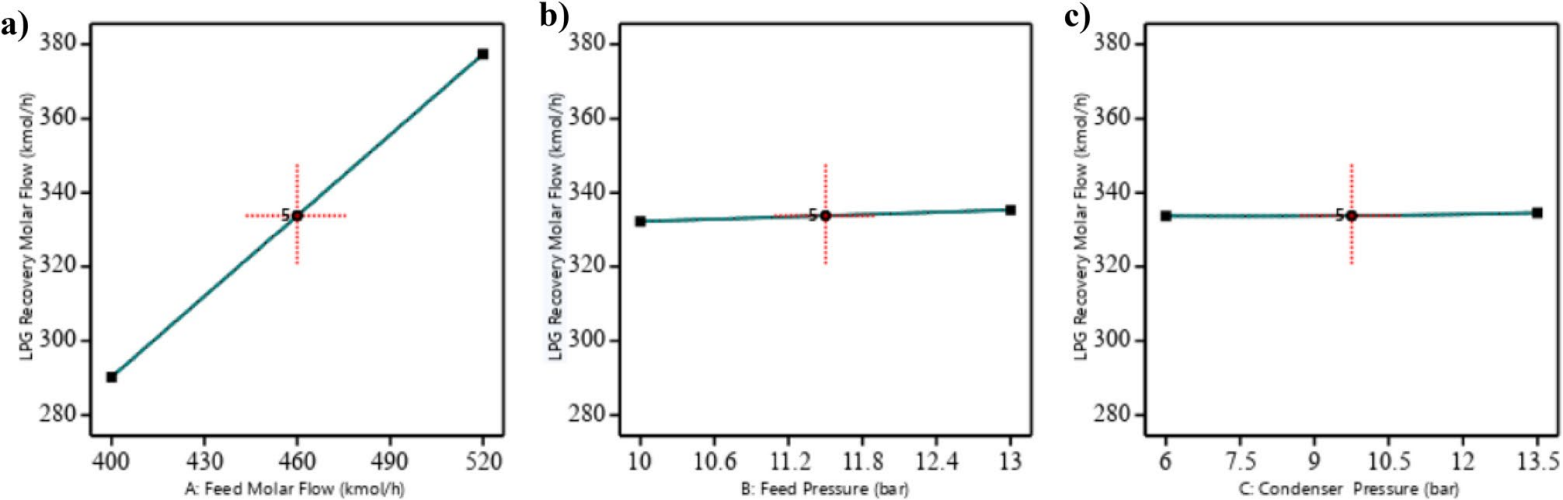
Main effect plots of operating parameters on LPG recovery molar flow in the debutanizer column, showing the individual influence of (**a**) feed molar flow rate (400–520 kgmole/h), (**b**) feed pressure (10–13 bar), and (**c**) condenser pressure (6–13.5 bar).
2. Effect of condenser pressure on the response variable.
  Condenser pressure has a significant positive linear effect (p-value < 0.0001), with a coefficient of 0.425, and a significant quadratic effect (p-value < 0.0001), with a coefficient of 0.3525 for C^2^, indicating a non-linear relationship. The RSM plot (Figs. 8 and 9) shows that LPG recovery increases with condenser pressure from 6 to 13.5 bar, with the surface sloping upward. The color gradient (blue to red) and red dots (e.g., at 380 kgmole/h, 520 kgmole/h feed molar flow, 13.5 bar condenser) suggest that recovery peaks at higher condenser pressures.
  The individual effect plot for condenser pressure (Fig. 11c) shows a nearly horizontal line at approximately 333 kgmole/h across condenser pressure from 6 to 13.5 bar, indicating a negligible main effect, with a change of about 0.85 kgmole/h, which is hard to notice given the scale. This aligns with the observation that the increase is slight and hard to notice, despite statistical significance.
  In conclusion, the interaction between feed pressure and condenser pressure (Fig. 10) does not significantly affect LPG recovery, with a p-value of 1.00, indicating no additional impact beyond their individual effects. This means varying both pressures together don’t lead to noticeable changes in recovery. Also, Fig. 11b and c shows that feed pressure and condenser pressure show only slight increases in LPG recovery, with changes of about 3.075 kgmole/h and 0.85 kgmole/h, respectively, from low to high levels. These small changes are hard to notice in plots, as they remain nearly constant at around 330–340 kgmole/h, compared to the significant increase seen with feed molar flow rate (Fig. 11a). Increasing the system pressure, especially at the condenser, enhances LPG recovery due to its effect on the vapor-liquid equilibrium (VLE). At higher pressures, the VLE shifts in favor of the liquid phase, increasing the condensation rate of heavier hydrocarbons such as propane and butane. This results in a higher recovery rate, as more desired components are driven into the liquid phase. Additionally, elevated condenser pressure raises the dew point temperature, improving phase separation efficiency and reducing vapor losses.

### Process optimization

The optimization process was performed using Design Expert, where the objective was to maximize LPG recovery while considering engineering, operational, and economic constraints. Three key process variables were optimized: feed molar flow rate, feed pressure, and condenser pressure. Each variable was assigned an appropriate constraint based on practical considerations. The feeding molar flow rate was set to “in range” to ensure a balance between maximizing LPG recovery and minimizing raw material costs. The engineering constraints were represented through upper and lower bounds on key process variables such as feed molar flow rate, feed pressure, and condenser pressure, ensuring operation within safe and technically feasible limits. These constraints were embedded into the Design Expert^®^ environment through defined variable limits and optimization targets. Operational constraints were considered by maintaining practical control ranges particularly for the reflux ratio to support process stability and controller responsiveness. Economic considerations, including energy efficiency related to reboiler and condenser duties, were evaluated based on the simulation outcomes following optimization, ensuring that recovery enhancement did not compromise process economics.

Feed pressure was optimized with a “target” value to maintain system stability and avoid excessive energy consumption associated with high-pressure operation. Condenser pressure was designated as “minimize” to enhance LPG separation efficiency while preventing excessive refrigeration costs. Table 9 summarized the design constraints used in this study for LPG optimization.

**Table 9:** Optimization constraints and goals for process variables.

Name	Goal	Lower limit	Upper limit
Feed molar flow	Is in range	400	520
Feed pressure	Is in range	10	13
Condenser pressure	Minimize	6	13.5
LPG recovery molar flow	Maximize	288.9	379.1

The optimization process in Design Expert generated 61 potential solutions, each representing a combination of factor levels that maximize LPG recovery while meeting the set goals. To ensure high-quality outcomes, solutions with desirability below 0.95 were excluded, as these were deemed less optimal in balancing recovery, cost, and operational constraints. Desirability, a composite score from 0 to 1, reflects the trade-off between all objectives, with higher values indicating better overall performance.

**Fig. 12:**
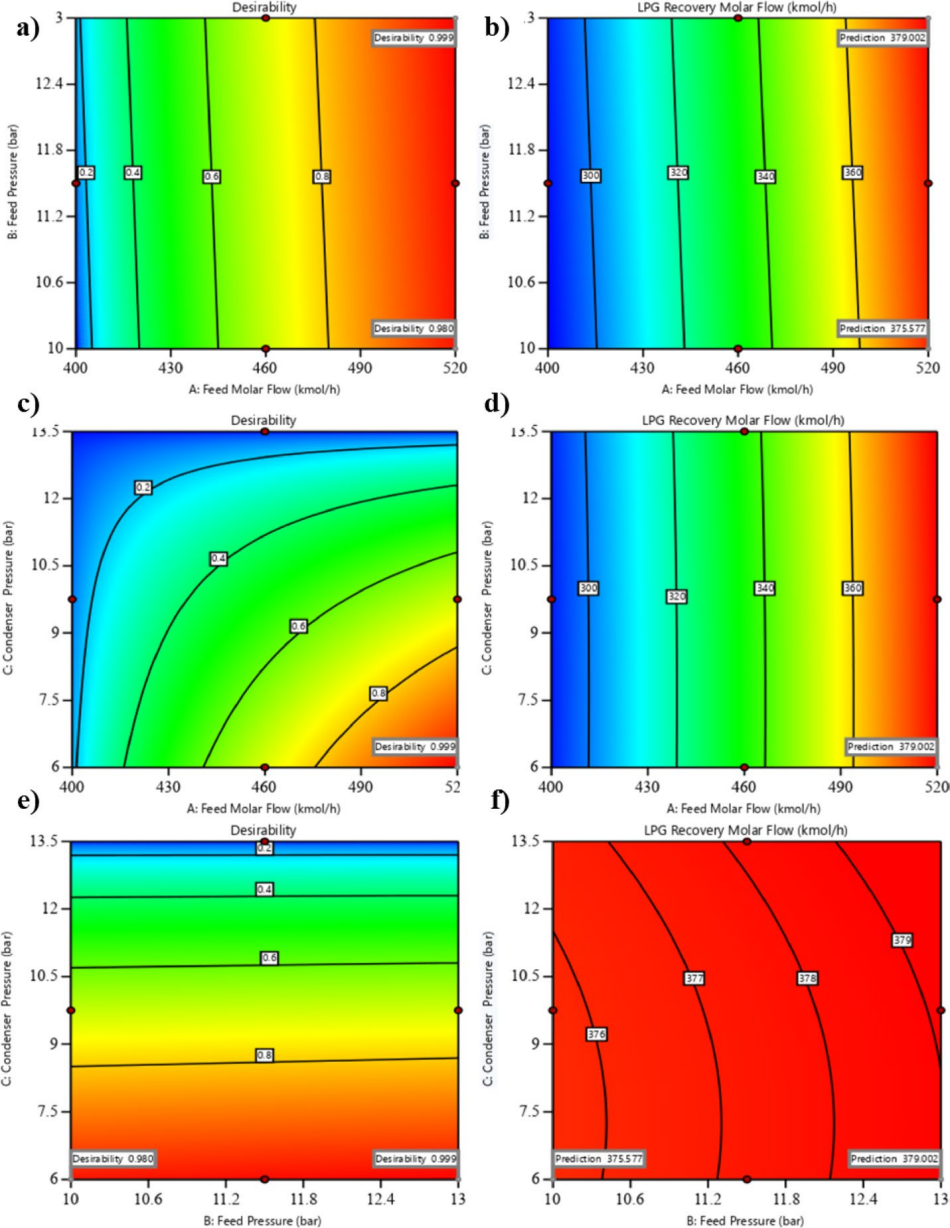
Optimization contour plots for maximizing LPG recovery in the debutanizer column using Design Expert. Subplots (**a**), (**c**), and (**e**) show desirability contours for the interactions between feed molar flow rate and feed pressure, feed molar flow rate and condenser pressure, and feed pressure and condenser pressure, respectively, with desirability values ranging from 0.2 to 0.999 and optimal points marked at 0.999. Subplots (**b**), (**d**), and (**f**) display predicted LPG recovery molar flow (kgmole/h) contours for the same variable pairs, with recovery values ranging from 300 to 379.002 (B and D) and 375.577 to 379.002 (F), and optimal points marked at 379.002 kgmole/h.

The final selection was the solution with the highest desirability, which balanced the maximization of feed molar flow rate within equipment limits, targeting an optimal feed pressure to minimize compression costs, and minimizing condenser pressure within a practical range to enhance recovery without excessive refrigeration costs. The selected optimal conditions were a feed molar flow rate of 520, a feed pressure of 13, and a condenser pressure of 6, resulting in an LPG recovery molar flow of 397.002. This solution ensured feasibility in industrial applications, considering engineering constraints such as column capacity, compressor limits, and cooling system capacity, as well as economic constraints like energy and raw material costs. The detailed optimization results are illustrated in Fig. 12.

Figure 12 presents six contour plots (a–f) illustrating the desirability and predicted LPG recovery molar flow across various combinations of key process variables: feed molar flow rate, feed pressure, and condenser pressure. These plots provide insight into the optimization landscape and highlight the optimal conditions for maximizing LPG recovery. Subplot (a) depicts a contour plot of feed molar flow rate (400–520 kgmole/h) against feed pressure (10–13 bar), with desirability contours ranging from 0.2 to 0.999. The optimal operating point, corresponding to a desirability of 0.999, is clearly marked. Similarly, subplot (b) presents the corresponding predicted LPG recovery molar flow, where the same axes are used, and recovery values range from 300 to 379.002 kgmole/h. The optimal condition, achieving a predicted LPG recovery of 379.002 kgmole/h, is highlighted. Subplot (c) displays a feed molar flow rate vs. condenser pressure (6–13.5 bar) contour plot for desirability, following the same trend as subplot (a), with an optimal desirability of 0.999 marked. Meanwhile, subplot (d) illustrates the predicted LPG recovery molar flow for feed molar flow rate vs. condenser pressure, showing values between 300 and 379.002 kgmole/h, with the optimal recovery condition indicated. Subplot (e) examines the interplay between feed pressure and condenser pressure, displaying desirability contours, again ranging from 0.2 to 0.999, with the optimal desirability of 0.999 clearly identified. Finally, subplot (f) provides the predicted LPG recovery molar flow in the feed pressure vs. condenser pressure space, with recovery values ranging from 375.577 to 379.002 kgmole/h. The optimal point, yielding 379.002 kgmole/h, is distinctly marked. These graphical representations facilitate the visualization of optimal operating conditions and the trade-offs among process variables, reinforcing the robustness of the selected optimization strategy. While the desirability-based contour plots effectively identify optimal steady-state operating conditions, their direct application in dynamic industrial environments poses challenges. Real-world processes often experience fluctuations and disturbances, which static optimization tools like the desirability function do not account for. Therefore, future work should focus on integrating dynamic desirability-based optimization within real-time control systems, allowing more adaptive and robust decision-making under varying operational conditions.

### Identification of the disturbance variable

An interesting finding is the distinction between statistical and practical significance. While feed pressure and condenser pressure are statistically significant due to the precision of simulation data, their small effect sizes (coefficients 1.5375 and 0.425) mean their practical impact on LPG recovery is limited compared to feed molar flow rate. This highlights the importance of considering both statistical and real-world relevance in process control, especially for high-impact journal publications.

For the purpose of MPC, the most influential factor is selected as the disturbance variable, so feed molar flow rate should be considered the primary disturbance variable due to its dominant effect on LPG recovery. Feed pressure and condenser pressure, while statistically significant, have practical impacts that are minor, suggesting they can be treated as secondary variables or manipulated variables within the control framework.

### MPC controller performance and recovery enhancement

**Fig. 13:**
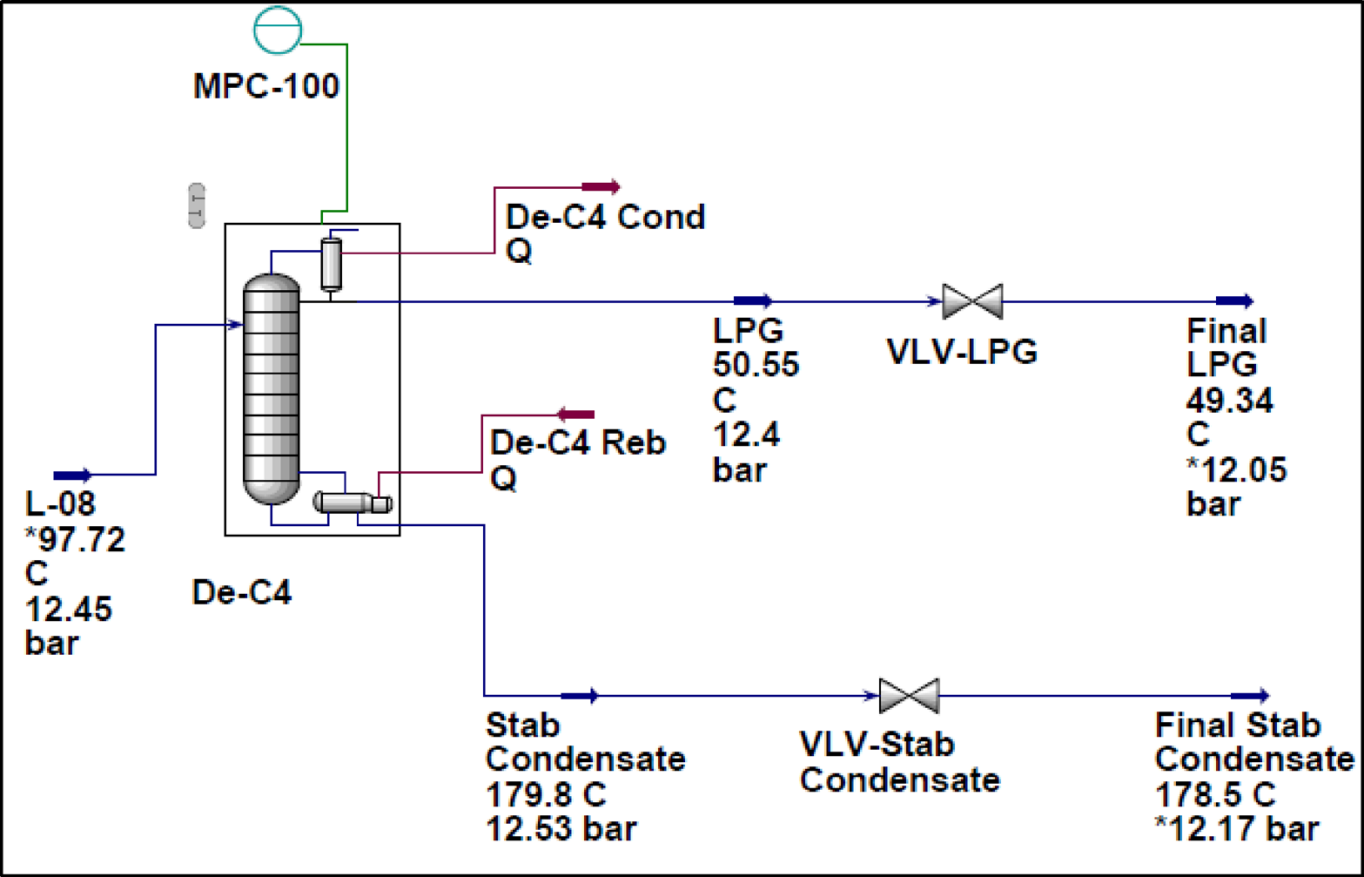
MPC simulation printout of debutanizer unit.

**Fig. 14:**
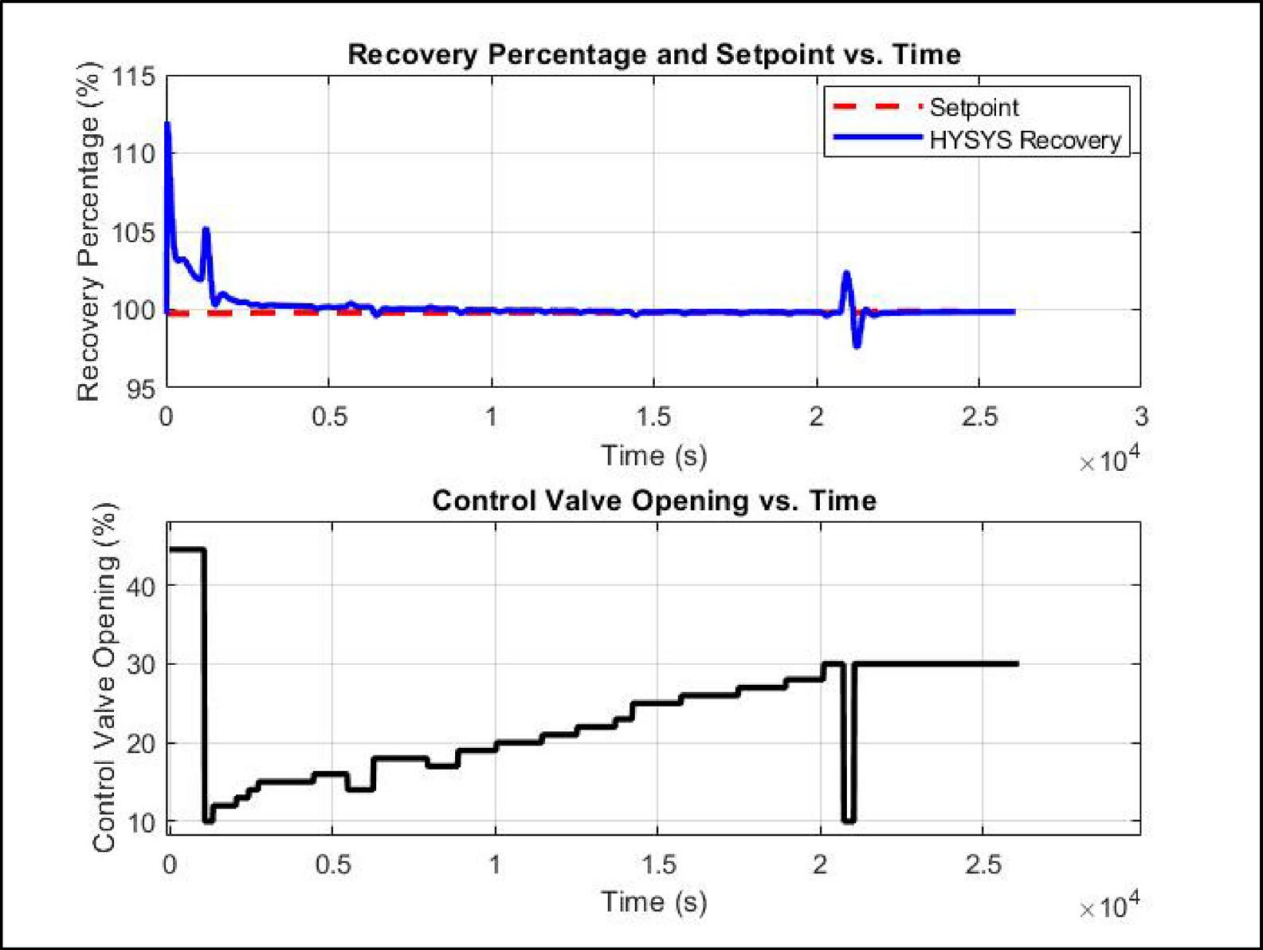
MPC simulation script chart printout of debutanizer unit.

The implementation of MPC led to substantial enhancements in LPG recovery, process stability, and setpoint tracking. The controller effectively minimized fluctuations, ensuring that the reflux flow rate remained within the optimal range and reducing unnecessary adjustments to the control valve. The control valve opening stabilized at 30%, maintaining steady-state conditions for an extended period, which contributed to improved process efficiency. Additionally, LPG pressure remained stable at approximately 12.05, preventing disturbances that could negatively impact product recovery. The dynamic simulation flowchart shown in Fig. 13 along with dynamic results illustrated in Fig. 14 demonstrate the superiority of MPC over traditional PID control in managing multivariable interactions and enforcing operational constraints. Unlike PID, MPC’s predictive capability allowed proactive adjustments, enhancing setpoint tracking and reducing energy consumption. During variations due to disturbances as shown in Table 10 like feed pressure, column pressures, overhead pressure and boil up flow rate, MPC maintained LPG recovery at 99.85%, with a molar flow rate of 365.5 kgmole/h. The recovery percentages of i-C_4_ and n-C_4_ increased to 99.91% and 99.19%, respectively, reflecting improved separation efficiency. Furthermore, energy efficiency was enhanced, as indicated by a decrease in reflux molar flow to 271 kgmole/h while maintaining stable condenser duty and slight decrease in reboiler duty at 2,493,000 kcal/h and 1,550,000 kcal/h, respectively. The temperature profile of LPG remained controlled at 49.34 °C, supporting stable operation. These results confirm that MPC not only ensured setpoint tracking but also optimized process performance by maximizing LPG recovery, reducing energy consumption, and maintaining stable operating conditions.

The MPC performance analysis in Fig. 14 demonstrates its superior ability to regulate LPG recovery percentage while optimizing control valve positioning over an extended operational period. Initially, at t = 0 s, the system started with a control valve opening of 44.53%, achieving an LPG recovery of 99.73%, closely aligning with the setpoint. By 1000 s, the recovery percentage slightly increased to 101.95%, while the valve position remained unchanged, reflecting the controller’s early-stage adjustments. At 3000 s, MPC actively optimized the process, reducing the valve opening to 15%, which stabilized the LPG recovery at 100.22%, preventing unnecessary energy consumption. As the operation progressed, at t = 5000 s, the control valve adjusted to 16%, maintaining a consistent recovery of 100.16%, demonstrating MPC’s capability to hold the process variable within a tight range. Further optimization occurred at t = 7000 s, with the valve opening set to 18%, ensuring that the recovery percentage closely matched the setpoint at 100.00%. By 10,000 s, fine-tuned adjustments brought the recovery to 99.97%, with the valve stabilized at 19%, highlighting MPC’s precise control actions. Long-term stability was evident at t = 15,000 s, where the recovery remained at 99.83% with a valve opening of 25%, followed by t = 20,000 s, where 99.84% recovery was sustained with the valve at 28%. Ultimately, at t = 26,120 s, the system reached full steady-state optimization, with the LPG recovery stabilizing at 99.85% and the control valve at 30%, showcasing MPC’s success in enhancing recovery beyond steady-state targets.

Unlike traditional PID control, which often requires frequent, large valve movements, MPC systematically optimized control actions, reducing energy consumption and minimizing mechanical wear. The ability to maintain LPG recovery within a precise range while stabilizing control valve adjustments confirms the effectiveness of MPC in improving process efficiency, ensuring minimal fluctuations, and achieving superior performance in debutanizer column operations.

**Table 10:** Main expected process disturbances and their influence on column performance.

Disturbance	Steady-state value	Dynamic value	Change	Impact on process
Feed pressure (bar)	13	12.45	−0.55	Affects relative volatility, impacting separation and product purity
Feed vapor fraction	0.6132	0.64	+ 0.0268	Changes vapor-liquid equilibrium, influencing column stability
Overhead pressure (bar)	12.39	12.05	−0.34	Reduced pressure may change component volatility, affecting LPG recovery
Overhead temperature (°C)	49.88	49.34	−0.54	Impacts fractionation efficiency, affecting propane purity
Bottom pressure (bar)	12.73	12.52	−0.21	It affects efficiency and energy consumption
Boil-up rate (kgmole/h)	272.5	260	−12.5	Reduction in vapor flow may impact stripping efficiency

**Fig. 15:**
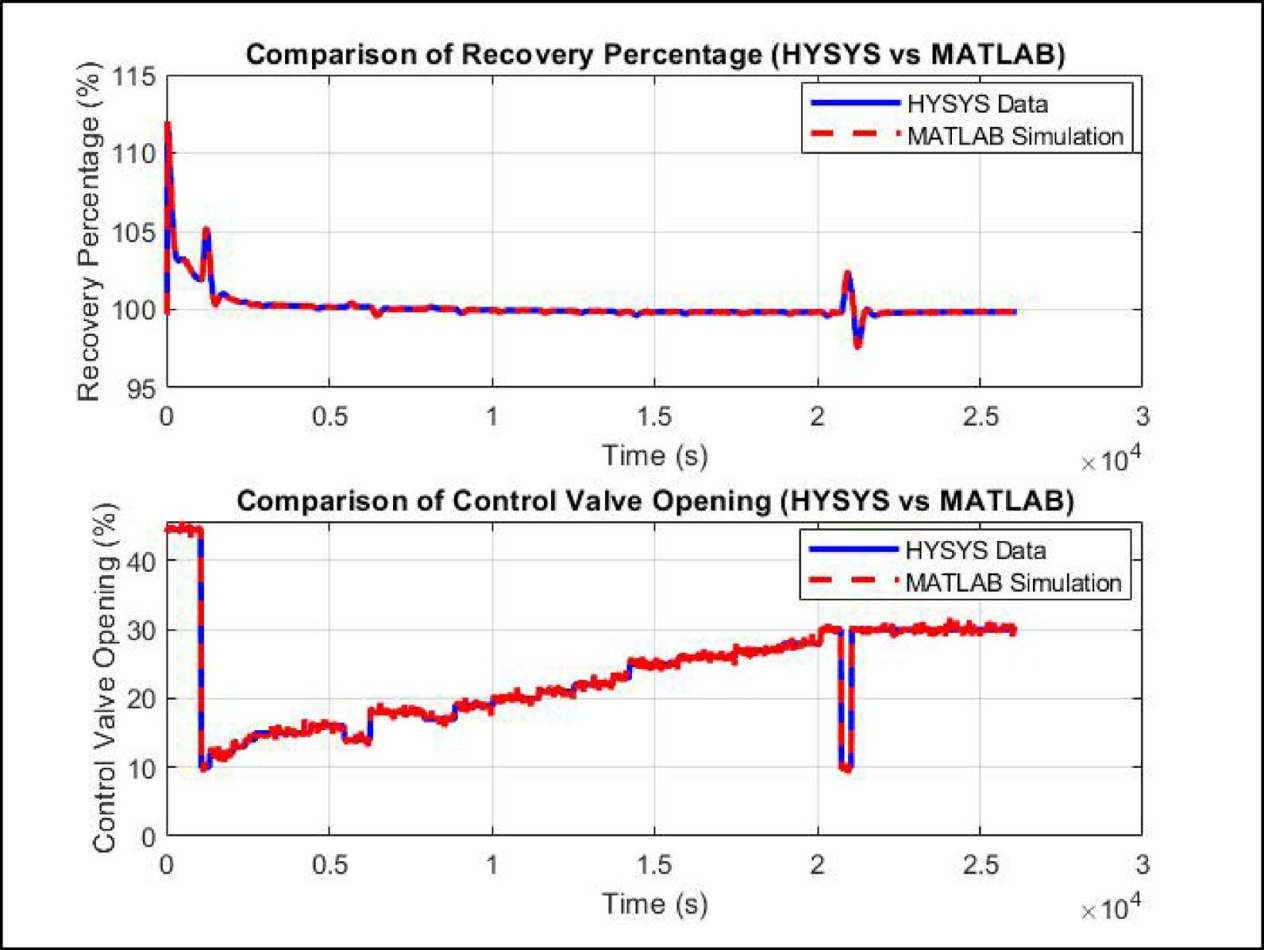
Comparative analysis of MPC performance: HYSYS vs. MATLAB simulation for LPG recovery control.

A comparative analysis of LPG recovery percentage and control valve opening over time between the HYSYS-based MPC simulation and its corresponding MATLAB simulation which has presented in Fig. 15. The recovery percentage exhibits an initial overshoot, reaching approximately 112% before gradually settling towards the setpoint of 99.73%. The system stabilizes around this value after t ≈ 5,000 s, with both MATLAB and HYSYS showing nearly identical responses. A minor fluctuation occurs at t ≈ 20,000 s, where the recovery percentage briefly deviates by approximately 0.5% before returning to steady-state. The control valve opening follows a similar trend in both simulations. Initially, the valve opens at ≈ 42%, followed by a rapid decrease to below 10%, after which it gradually increases in response to process requirements. A noticeable drop in valve opening occurs around 20,000 s, aligning with the observed fluctuation in recovery percentage. The high degree of correlation between MATLAB and HYSYS results indicate that the developed MPC model accurately replicates the dynamic behavior of the debutanizer column.

### AI-enhanced MPC performance

**Fig. 16:**
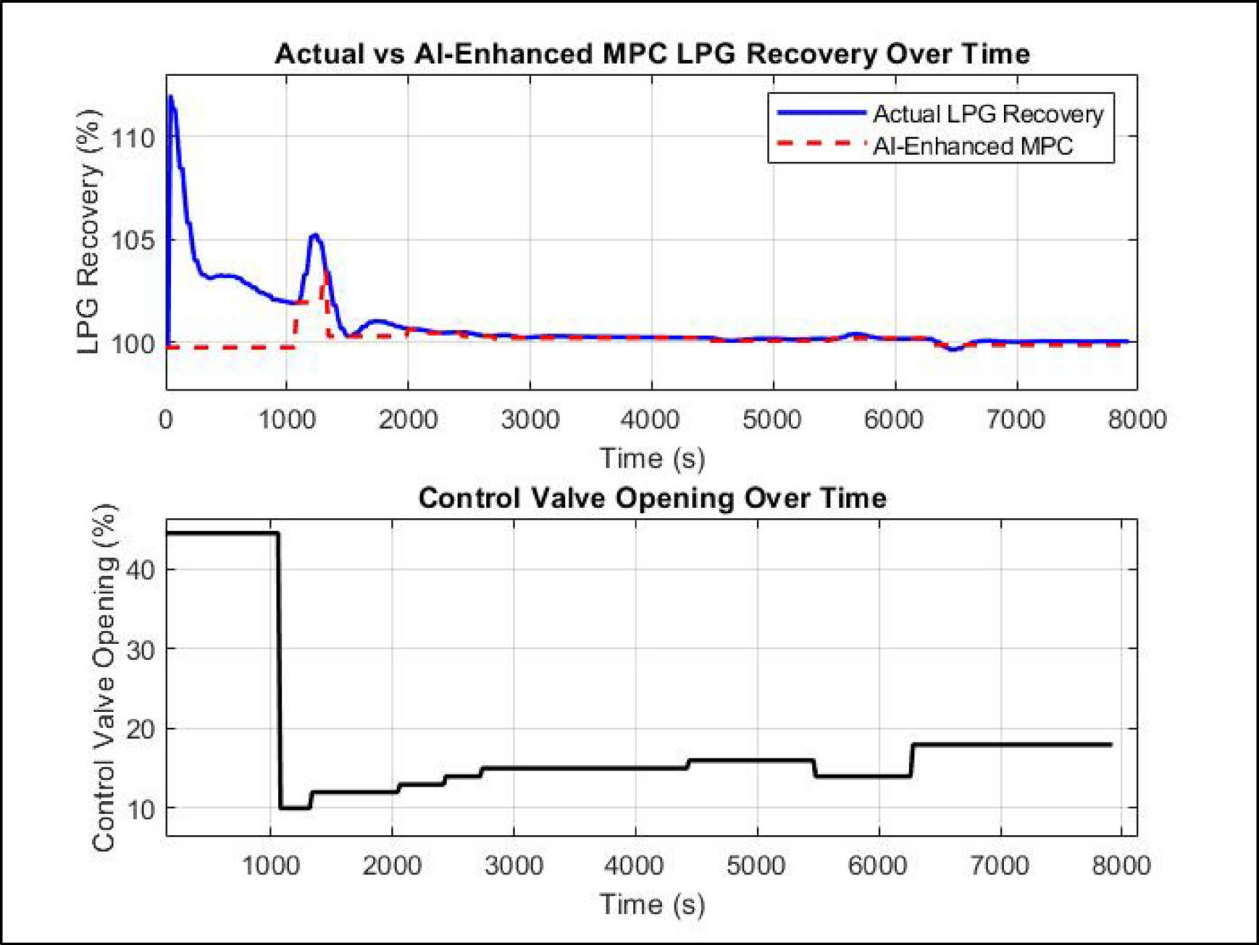
AI-enhanced MPC performance.

The performance of the AI-enhanced MPC in regulating LPG recovery percentage over time while adjusting the control valve opening is illustrated in Fig. 16. The first subplot presents the actual LPG recovery percentage in response to a setpoint of 99.85%, while the second subplot depicts the corresponding control valve opening behavior. The AI-enhanced MPC demonstrates a stable response, effectively maintaining the LPG recovery percentage close to the setpoint with an improvement. The more pronounced deviation between MPC and AI-enhanced MPC during the first ~ 1500 s can be attributed to the transient response period, where the AI-based controller responds faster and more efficiently to setpoint changes and initial disturbances. Once the system reaches steady-state conditions, both controllers converge to maintain the target recovery, explaining the minimal difference observed beyond 1500 s. Consequently, most of the energy savings due to optimized valve positioning and reduced control effort are realized during the transient phase, where AI-enhanced MPC outperforms traditional MPC in settling time and overshoot minimization.

Initially, the recovery percentage exhibits a peak overshot reaching approximately 112% within the first 200 s. This is followed by oscillations, with a second peak around 105% at approximately 1400 s. After 2000 s, the AI-enhanced MPC stabilizes the recovery percentage, maintaining it at 99.9% in a steady-state. The second subplot presents the control valve opening behavior. Initially, the valve opened at 45%, but it rapidly decreased to nearly 8% at around 500 s. The valve then undergoes gradual stepwise adjustments, reaching 12% at 2500 s and finally stabilizing at 18% after 6000 s. These results demonstrate the AI-enhanced MPC’s ability to effectively regulate LPG recovery, minimizing fluctuations and achieving a stable and improved recovery percentage from 99.85 to 99.9%. Unlike traditional MPC, which often exhibits significant overshoots (e.g., 112% at 400 s in HYSYS simulations), the AI-enhanced MPC exhibits a more gradual and controlled convergence towards the setpoint with smaller fluctuations, remaining within a ± 0.05% range after steady-state is achieved.

This suggests that the AI-enhanced MPC effectively maintains the desired recovery percentage with a significantly lower valve opening compared to MPC in HYSYS, which required 30% valve opening to achieve the same LPG recovery. This reduction in control valve opening translates to potential energy savings and improved process efficiency, as the AI-based approach optimizes valve positioning to maintain product recovery with minimal actuator movement. These findings confirm that AI-enhanced MPC can effectively regulate LPG recovery percentage with high accuracy while requiring a lower control effort, reducing energy consumption and valve wear. However, while the controller ensures precise recovery percentage regulation, further optimization may be required to minimize the minor oscillations observed in the control valve opening.

### A comparison of MPC and AI-enhanced MPC in LPG recovery performance

**Fig. 17:**
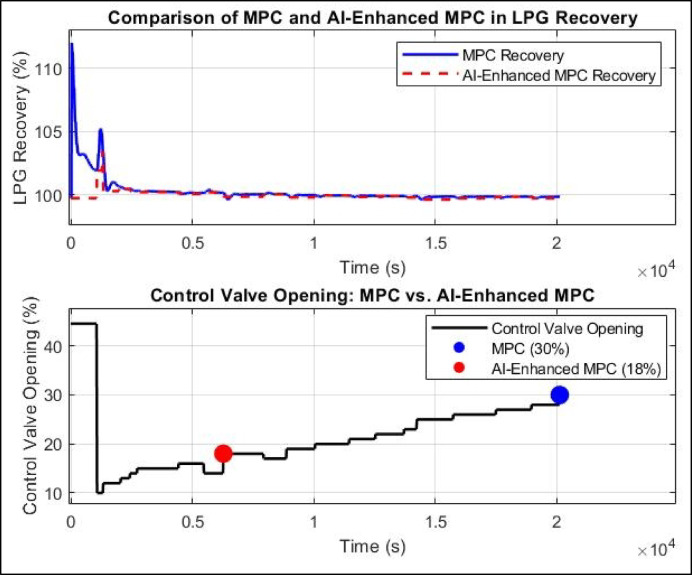
Performance comparison of traditional MPC vs. AI-enhanced MPC in LPG recovery control.

A critical comparison between the conventional MPC and AI-enhanced MPC in controlling LPG recovery percentage and control valve opening is presented in Fig. 17. The first subplot tracks the system’s ability to maintain LPG recovery at the setpoint 99.85% for MPC only and 99.9% for AI-enhanced MPC, while the second subplot monitors the control valve opening over time. The results reveal a striking contrast in performance, particularly in terms of response time, stability, and energy efficiency.

The MPC-only controller exhibits an initial overshoot, with the LPG recovery percentage spiking sharply to 112% at t = 400 s, before undergoing a prolonged settling phase. Despite eventually stabilizing near the setpoint, MPC requires approximately 26,020 s to reach a steady-state recovery of 99.85%. The control valve opening initially remains at 44.53%, but at t = 1080 s, it undergoes an abrupt reduction to 10%, leading to a temporary decline in LPG recovery before the system re-adjusts. The valve opening then gradually increases to 30% at t = 26,020 s, at which point the system stabilizes at 99.85% recovery. While the MPC effectively maintains the setpoint, the prolonged settling time and high control effort suggest suboptimal energy efficiency.

By contrast, the AI-enhanced MPC controller introduces a smoother and more adaptive response, significantly improving the system’s performance. The AI-enhanced MPC reaches the steady-state and improves LPG recovery to 99.9% much faster, at t = 19,860 s, reducing the settling time by approximately 6,160 s compared to traditional MPC. Furthermore, the control effort required for stabilization is notably lower, as the AI-augmented controller stabilizes the control valve opening at 18% from t = 7000 s onwards, whereas standard MPC requires a significantly higher 30% valve opening. This reduction of 40% in control valve opening translates directly to improved energy efficiency, as a lower valve opening minimizes reflux flow and reduces energy consumption.

The results clearly illustrate that AI enhances the performance of MPC by optimizing control actions, increasing LPG recovery percentage, minimizing settling time, and reducing valve variability. Unlike traditional MPC, which relies solely on predefined model predictions, the AI-enhanced controller adapts dynamically to process disturbances, leading to faster convergence (by 6,160s), reduced overshoot, and a more energy-efficient operation. The AI-enhanced approach also mitigates control valve oscillations, ensuring a smoother transition towards steady-state, which in turn reduces mechanical wear on the valve and improves process stability.

As summarized in Table 11, the AI-enhanced MPC significantly outperformed both steady-state and conventional MPC operations across all key performance indicators. These include higher LPG recovery and molar flow rate, improved component separation efficiency (C_3_, i-C_4_, n-C_4_), and reduced energy consumption, as indicated by lower condenser and reboiler duties. Furthermore, the AI-based controller optimized reflux flow and enhanced pressure and temperature stability, confirming its superiority in achieving efficient and stable LPG recovery under dynamic operating conditions.

**Table 11:** Comparison of results for steady-state, MPC and AI-enhanced MPC in the debutanizer unit.

Parameter	Control strategy		
	Steady-state (St St)	MPC controller	AI-enhanced MPC
LPG recovery %	99.73%	99.85%	99.9%
C_3_ recovery %	100%	100%	100%
i-C_4_ recovery %	99.87%	99.91%	99.90%
n-C_4_ recovery %	98.48%	99.19%	99.50%
LPG molar flow (kgmole/h)	360.4	365.5	372.81
Condenser duty (kcal/h)	2,493,000	2,493,000	2,415,726
Reboiler duty (kcal/h)	1,557,000	1,550,000	1,501,956
Reflux molar flow (kgmole/h)	281.2	271	262.6
Temperature (°C)	49.88	49.34	49.24
Pressure (bar)	12.39	12.05	11.95

The observed reductions in reboiler and condenser duties, along with a decrease in the reflux flow rate, indicate significant energy savings. These improvements enhance overall process efficiency, particularly in large-scale operations where cumulative reductions in thermal load and utility demands are critical for sustainable performance. The comparative analysis confirms that integrating intelligent control mechanisms significantly enhances LPG recovery performance. While conventional MPC improved both recovery and energy usage, achieving a recovery of 99.85%, the AI-enhanced approach achieved further gains with a consistent recovery of 99.90%, while also reducing condenser and reboiler duties and lowering reflux flow rates It maintained this performance using a smaller valve opening (18%) compared to 30% with MPC, indicating better actuator efficiency and reduced control effort. These enhancements were obtained while maintaining overall process stability, highlighting the benefit of incorporating AI into advanced control strategies for dynamic recovery systems. A detailed quantitative comparison reveals the performance gains achieved by each control strategy. The AI-enhanced MPC improved LPG recovery from 99.73% under steady-state conditions to 99.90%, representing an overall improvement of 0.17%. Compared to conventional MPC (99.85%), it provided an additional gain of 0.05% in recovery. In terms of energy efficiency, the reboiler duty was reduced by 3.1% (from 1,550,000 to 1,501,956 kcal/h), and condenser duty decreased by 3.1% (from 2,493,000 to 2,415,726 kcal/h). Moreover, the AI-based controller required only 18% valve opening to achieve the same or better performance, compared to 30% with MPC indicating a 40% reduction in control effort. These numerical comparisons confirm the superiority of AI-enhanced MPC in both recovery enhancement and energy optimization.

The reduction in product temperature and pressure reflects the direct influence of AI-enhanced MPC in stabilizing the column’s internal conditions. While these values do not directly affect VLE, they indicate tighter control over the thermal profile, reduced oscillations, and improved phase stability, which together enhance separation efficiency and product consistency.

To further demonstrate the practical significance of the proposed control strategy, the observed improvement in LPG recovery was accompanied by enhanced process stability and consistent product behavior. While some previous studies focused primarily on optimizing steady conditions, relying solely on steady-state modeling without dynamic control implementation, or applying deep learning and PSO-based optimization frameworks limited to steady-state parameter tuning without any real-time or dynamic control structure^37,47^. On the other hand, another study extended their work beyond steady-state analysis by implementing dynamic control using conventional PID and MPC techniques; however, their approach did not incorporate any AI-based adaptation or advanced learning structures, which distinguishes the present study^48^. The selected operating parameters for current work, particularly feed flow rate and condenser pressure, contributed to better phase separation and reduced variability in reflux flow and valve response. These factors collectively improved the separation consistency, which is directly linked to the quality and reliability of the overhead product. Although the increase in recovery appears small in absolute value, it reflects a measurable improvement in controllability and overall efficiency, achieved without introducing major changes to process design or operating conditions.

## Conclusion

This study presents a systematic approach to optimize LPG recovery in debutanizer columns through advanced control strategies. Using Response Surface Methodology with Box-Behnken Design, we identified feed molar flow rate as the most significant factor affecting recovery (*p* < 0.0001), while feed and condenser pressures showed lesser impact. A highly accurate quadratic model (R^2^ = 1.0000) was developed to predict system behavior. The research compared three operational modes: steady-state, conventional MPC, and AI-enhanced MPC. While steady-state operation achieved 99.73% recovery, MPC improved this to 99.85% with reduced energy consumption (reboiler duty: 1,550,000 kcal/h) and reflux flow (271 kgmole/h). The AI-enhanced MPC demonstrated superior performance, reaching 99.9% recovery while further lowering energy requirements (reboiler: 1,501,956 kcal/h; condenser: 2,415,726 kcal/h) and reflux flow (262.6 kgmole/h). The key innovation lies in embedding a regression-based AI model within the MPC loop, allowing the controller to adapt in real time to system disturbances and nonlinearities-capabilities that conventional MPC lacks. This adaptive intelligence resulted in more stable operation and smoother control actions. These outcomes validate the added value of AI integration and position AI-enhanced MPC as a forward-looking strategy for complex industrial control systems. Future work could focus on further improving the adaptability of AI models to broader operational conditions and validating their application at larger industrial scales. Addressing integration challenges and ensuring efficient computational implementation will be essential for facilitating the widespread adoption of AI-enhanced control systems.

## Data Availability

Data Availability The datasets generated and analyzed during this study are available from the corresponding author upon reasonable request. Additionally, the Aspen HYSYS simulation files used in this research can be provided to facilitate further analysis or replication of the study.
